# Phospholipid Remodeling and Tri‐Layer Membrane Reconstruction Mediate Cognitive Effects of Humanized Milk Fat Globules in Neonatal Rats

**DOI:** 10.1002/advs.202507926

**Published:** 2025-09-14

**Authors:** Shaolei Wang, Fengzhi Qiao, Jian He, Jianxin Fu, Tongjie Liu, Huaxi Yi, Qinghai Sheng, Lanwei Zhang, Kai Lin

**Affiliations:** ^1^ State Key Laboratory of Marine Food Processing & Safety Control College of Food Science and Engineering Ocean University of China Qingdao 266003 China; ^2^ National Center of Technology Innovation for Dairy Hohhot 010000 China; ^3^ College of Food Science and Technology Hebei Agricultural University Baoding 071001 China

**Keywords:** Cognition, Humanized milk fat globules, Neurodevelopment, Phospholipid composition, Tri‐layer membrane

## Abstract

Human milk fat globules (MFG) are critical for cognitive development during early life. Here, the first study is presented to simulate the phospholipid composition of human milk fat globules and reconstruct their tri‐layer membrane to evaluate their effects on cognitive function. Humanized milk fat globule membrane phospholipids (MFGM‐PL+SM) significantly promoted the proliferation and neuronal differentiation of hippocampal neural progenitor cells (NPC). Transcriptomic and proteomic analyses reveal that MFGM‐PL+SM upregulated cholinergic signaling and neurotrophic factor pathways in differentiated NPC. Layer‐by‐layer deposition produced tri‐layer milk fat globules (T‐MFG) with enhanced stability and digestibility over single‐layer milk fat globules (S‐MFG). Formula supplementation with humanized MFG modulated serum metabolite profiles in rat pups, improving neurodevelopment and increasing hippocampal sphingomyelin levels. Moreover, key neurodevelopmental proteins in formula‐fed pups approached levels observed in breastfed controls. These findings highlight the potential of humanized MFG to support infant cognitive development and provide insights for designing enriched infant formulas.

## Graphical abstract

Humanized milk fat globules (MFG) mimic the native composition and structure of human milk, achieved through remodeling of milk fat globule membrane phospholipid composition and reconstruction of the tri‐layer membrane structure. These humanized MFG exhibit improved structural stability and digestibility, and enhance neurodevelopment in formula‐fed rat pups, supporting their potential to optimize infant formula for promoting cognitive development.

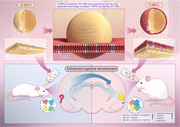

## 1 Introduction

Human milk (HM) is widely recognized as the optimal source of nutrition for infants, providing essential nutrients and influencing cognitive development through its complex lipid composition.^[^ ^1^, ^2^, ^3^ ^]^ Human milk fat globules (MFG) are primarily present as emulsions, supplying ≈50–60% of the energy required for infant growth and development.^[^ ^4^ ^]^ Each MFG is surrounded by a trilayer membrane structure known as the milk fat globule membrane (MFGM), which is rich in phospholipids and proteins. ^[^ ^5^ ^]^ Critical structural attributes of MFG, such as globule size, surface properties, and lipid composition, play vital roles in lipid digestion, absorption, and metabolism.^[^ ^6^, ^7^, ^8^ ^]^ In contrast, fat globules in infant formula are primarily coated with casein, leading to structural differences compared to human MFG.^[^ ^9^, ^10^ ^]^ These differences can influence lipid bioavailability and associated physiological functions.^[^ ^9^, ^11^ ^]^ Therefore, a thorough understanding of the unique structure of MFG in HM is essential for developing infant formulas that more closely mimic the structural and functional characteristics of natural milk.

Recent studies have increasingly highlighted the role of the gut‐brain axis in neurodevelopment. This bidirectional communication network links the gastrointestinal tract and the central nervous system through neural, immune, and metabolic pathways .^[^ ^12^, ^13^ ^]^ Its function is influenced by diet, gut microbiota, and host physiology. Lipids in HM not only provide a crucial source of energy but also influence the composition and activity of the gut microbiota, which may, in turn, affect neurodevelopmental and cognitive outcomes .^[^ ^12^, ^13^ ^]^ Reconstructed MFG significantly improve lipid bioavailability and functionality by converting milk fat into smaller, more uniform structures, thereby increasing lipid surface area.^[^ ^6^, ^9^, ^14^ ^]^ However, conventional reconstruction techniques generally produce single‐layered fat globules that differ structurally from the natural tri‐layered membranes found in humanized MFG. This structural difference is crucial for understanding lipid digestion and absorption, and presents new opportunities for exploring potential cognitive benefits.^[^ ^10^ ^]^ Despite these advances, the mechanisms by which reconstructed MFG enhance lipid utilization and support neurodevelopment, and how these processes contribute to improved cognitive function in infants, remain underexplored.

**Scheme 1:**
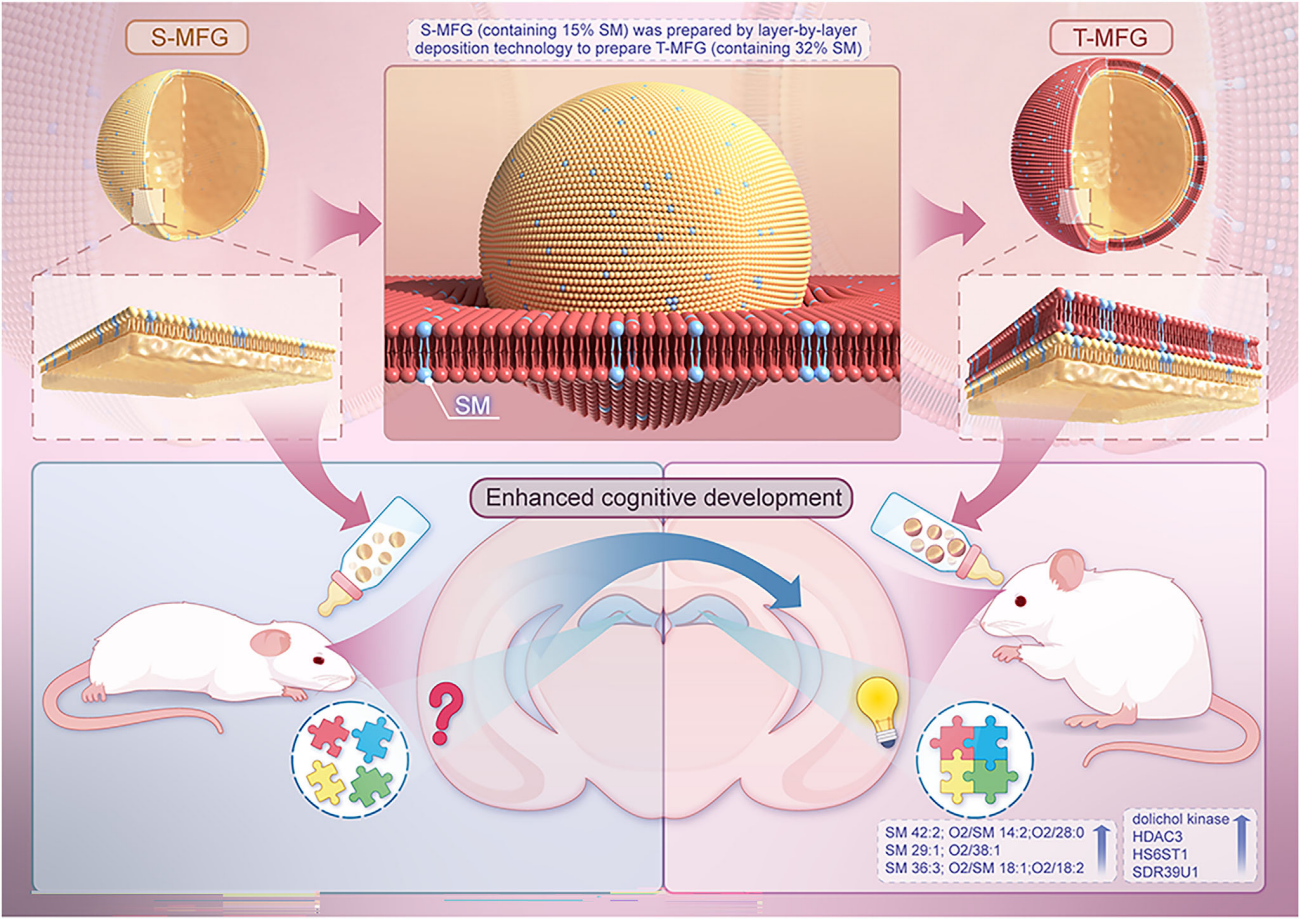
Schematic illustration showing the construction of humanized milk fat globules (MFG) and their effects on cognitive function. To generate tri‐layer MFG (T‐MFG), single‐layer MFG (S‐MFG) was further coated using a layer‐by‐layer deposition technique incorporating additional sphingomyelin (SM), thereby reconstructing a tri‐layer membrane structure enriched in SM to mimic the native architecture of human milk MFG. The humanized MFGs were orally administered to neonatal rats to assess their influence on brain development. The results demonstrated that humanized MFG (T‐MFG) enhanced neurodevelopment through: (1) improved performance in cognitive reflex tests; (2) increased hippocampal neuronal density and astrocyte proportion; (3) modified serum and hippocampal lipid profiles, particularly elevating SM and synapse‐associated lipids; (4) upregulation of proteins involved in neuronal growth and cognitive function; and (5) enrichment of gut microbiota associated with neurodevelopment.

Cow's milk is the most commonly used base for infant formula. In our previous study, significant differences were observed between the lipid compositions of cow and human MFGMs, with sphingomyelin (SM) accounting for 32% of lipids in human MFGM, which was significantly higher than the 15% observed in cow MFGM.^[^ ^15^ ^]^ Based on these findings, the present study investigated the regulatory effects of a humanized MFGM phospholipid composition on the proliferation and differentiation of hippocampal neural progenitor cells (NPC). A humanized MFG was engineered using a layer‐by‐layer deposition technique that incorporated two key elements: remodeling of MFGM lipid composition and reconstruction of the native tri‐layer membrane structure. The effects of the humanized MFG on lipid digestion and cognitive function were systematically evaluated in rat pups (**Scheme** **1**). This study addressed critical knowledge gaps in understanding the structural and functional roles of humanized MFG, establishing a theoretical basis for optimizing infant formula design. The findings examine whether humanized MFG can enhance cognitive function by promoting nutrient utilization and neurodevelopment. These insights contribute to the advancement of HM lipid–based formula development and support further research into the functional benefits of HM.

## 2 Results

### 2.1 Humanized MFGM Phospholipid Composition Promotes Hippocampal NPC Proliferation and Differentiation

**Figure 1:**
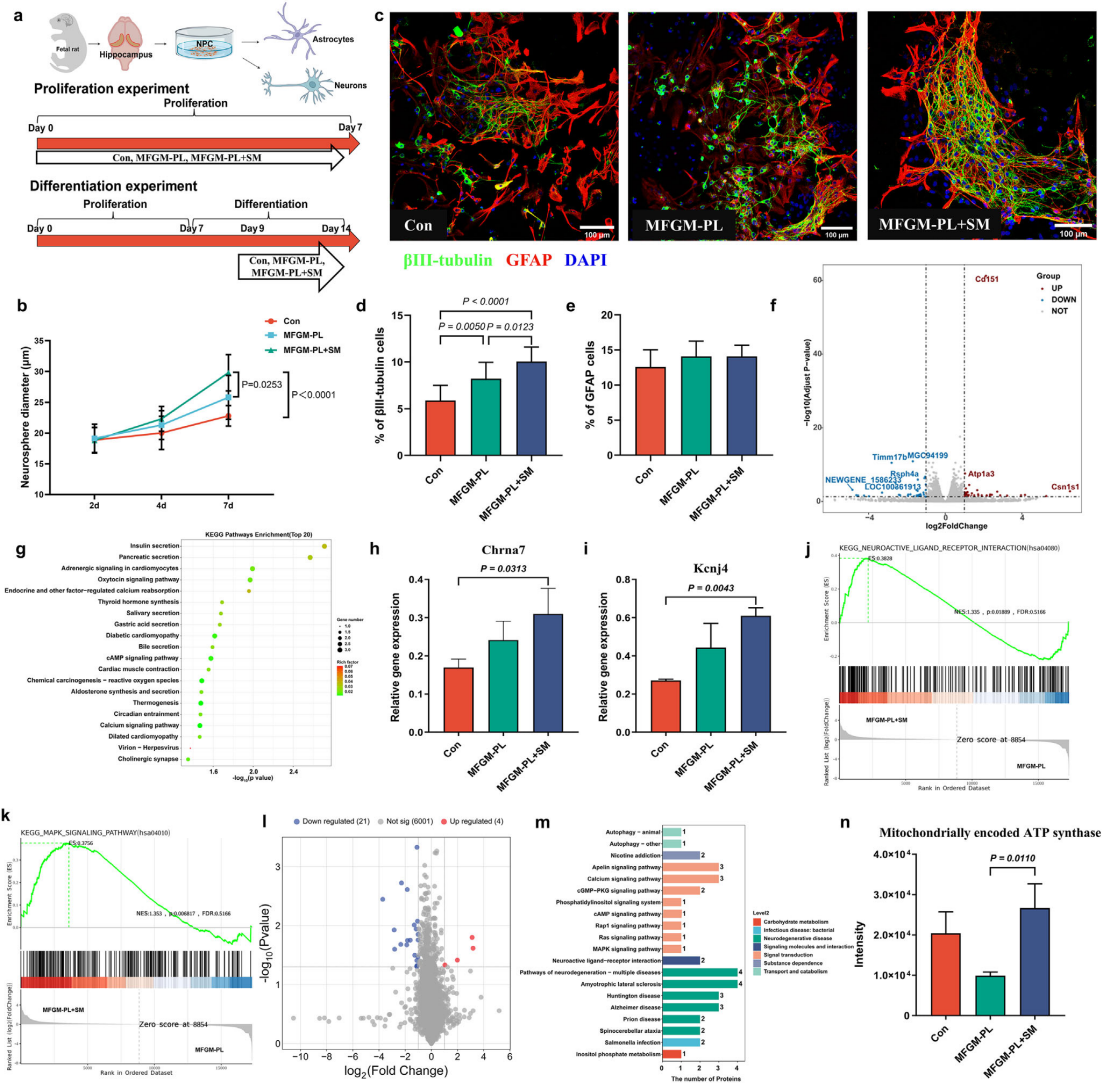
Humanized milk fat globule membrane phospholipid (MFGM‐PL+SM) composition enhances the proliferation and differentiation of hippocampal neural progenitor cells (NPC). a) Schematic illustration of the in vitro experimental design. Based on prior lipidomics analysis, a humanized MFGM phospholipid composition was reconstructed by increasing sphingomyelin (SM) content to match levels found in human milk (HM). Hippocampal NPC were cultured and treated with MFGM‐PL or MFGM‐PL+SM for subsequent assessments. b) MFGM‐PL+SM promotes neurosphere growth. Neurosphere diameter was significantly increased in the MFGM‐PL+SM group compared to the MFGM‐PL group after 7 days of culture. c–e) Immunofluorescence‐based analysis of NPC differentiation. βIII‐tubulin (green fluorescence) and glial fibrillary acidic protein (GFAP) (red fluorescence) staining were used to assess neuronal and astrocyte differentiation, respectively. Scale bar = 100 µm. MFGM‐PL+SM significantly enhanced neuronal differentiation, while astrocyte differentiation remained unaffected. f) Volcano plot showing differentially expressed genes (DEGs) between MFGM‐PL+SM and MFGM‐PL groups based on RNA sequencing after 5 days of differentiation. g) KEGG pathway enrichment analysis illustrating pathways associated with differentially expressed genes in the MFGM‐PL and MFGM‐PL+SM groups. h,i) Relative expression of *Kcnj4* and *Chrna7*, both significantly upregulated in NPC treated with MFGM‐PL+SM. j,k) Gene Set Enrichment Analysis (GSEA) showing the upregulation of neuroactive ligand‐receptor interaction and MAPK signaling pathways following treatment with MFGM‐PL+SM. l) Volcano plot showing differentially expressed proteins between MFGM‐PL+SM and MFGM‐PL groups identified by LC‐MS/MS‐based proteomics. Red dots indicate significantly upregulated proteins; blue dots indicate significantly downregulated proteins. m) KEGG pathway analysis of proteins significantly changed in NPC treated with MFGM‐PL+SM compared to MFGM‐PL. n) Relative protein expression of mitochondrially encoded ATP synthase, significantly upregulated in NPC treated with MFGM‐PL+SM compared to MFGM‐PL.

Based on our previous findings, the phospholipid composition of the MFGM differed significantly between human and cow milk, especially in SM content. SM accounts for ≈ 32% of total MFGM lipids in human milk, compared to only 15% in cow milk .^[^ ^15^ ^]^ To investigate the biological significance of this difference, a humanized MFGM phospholipid composition was developed by increasing the SM content to levels comparable to those found in human milk. The effects of different concentrations of MFGM phospholipids (MFGM‐PL) and humanized MFGM phospholipids enriched in SM (MFGM‐PL+SM) on NPC cytotoxicity, proliferation, differentiation, and transcriptomic and proteomic profiles were systematically evaluated (**Figure** **1**a). NPC were treated with MFGM‐PL or MFGM‐PL+SM for 24 h to assess cytotoxicity. Cell viability was not affected at any of the tested concentrations (Figure S1a, Supporting Information). Neurosphere diameter was used as an indicator of NPC proliferation. No significant differences were observed on day 2 (Figure S1b, Supporting Information). However, by days 4 and 7, the MFGM‐PL+SM groups (15 and 30 µg mL^−1^) showed significantly larger neurospheres. Furthermore, treatment with 30 µg mL^−1^ MFGM‐PL+SM resulted in a significant increase in neurosphere size after 7 days of in vitro culture compared to the MFGM‐PL group, indicating that MFGM‐PL+SM significantly enhanced NPC proliferation (Figure 1b). Immunofluorescence analysis revealed changes in the expression of βIII‐tubulin and glial fibrillary acidic protein (GFAP) following treatment with MFGM‐PL or MFGM‐PL+SM (Figure 1c). Both treatments promoted NPC differentiation into neurons, with a more pronounced increase in βIII‐tubulin expression observed in the MFGM‐PL+SM group (Figure 1d). In contrast, both MFGM‐PL and MFGM‐PL+SM had no significant effect on NPC differentiation into astrocytes (Figure 1e).

To further elucidate the molecular mechanisms underlying the effects of MFGM‐PL+SM on NPC differentiation, transcriptomic and proteomic analyses were performed after five days of differentiation. Transcriptomic analysis identified 73 differentially expressed genes (DEGs) between the MFGM‐PL+SM and MFGM‐PL groups (*P* < 0.05, fold change (FC) ≥ 2), including 33 downregulated and 40 upregulated genes in the MFGM‐PL+SM group (Figure 1f). KEGG pathway enrichment analysis revealed that these DEGs were involved in cognition‐related pathways, specifically the cholinergic synapse signaling pathway (Figure 1g). Notably, the expression levels of *Kcnj4* and *Chrna7*, two genes within this pathway, were significantly upregulated in the MFGM‐PL+SM group compared to the control group (Figure 1h,i). Gene Set Enrichment Analysis (GSEA) showed that MFGM‐PL+SM upregulated several cognition‐related signaling pathways, including the MAPK signaling pathway and neuroactive ligand‐receptor interaction pathway (Figure 1j,k). Proteomic analysis revealed that there were 25 differentially expressed proteins between the MFGM‐PL+SM and MFGM‐PL groups, including 21 downregulated and 4 upregulated proteins in the MFGM‐PL+SM group (Figure 1l). These proteins were primarily involved in signal transduction and neurodegenerative diseases (Figure 1m). Notably, the expression of mitochondrially encoded ATP synthase, a key marker of oxidative stress in neurodegenerative diseases, was significantly upregulated in the MFGM‐PL+SM group (Figure 1n).

### 2.2 Reconstruction of Humanized Milk Fat Globules and Their In Vitro Digestion Behavior Resembling Human Milk

**Figure 2:**
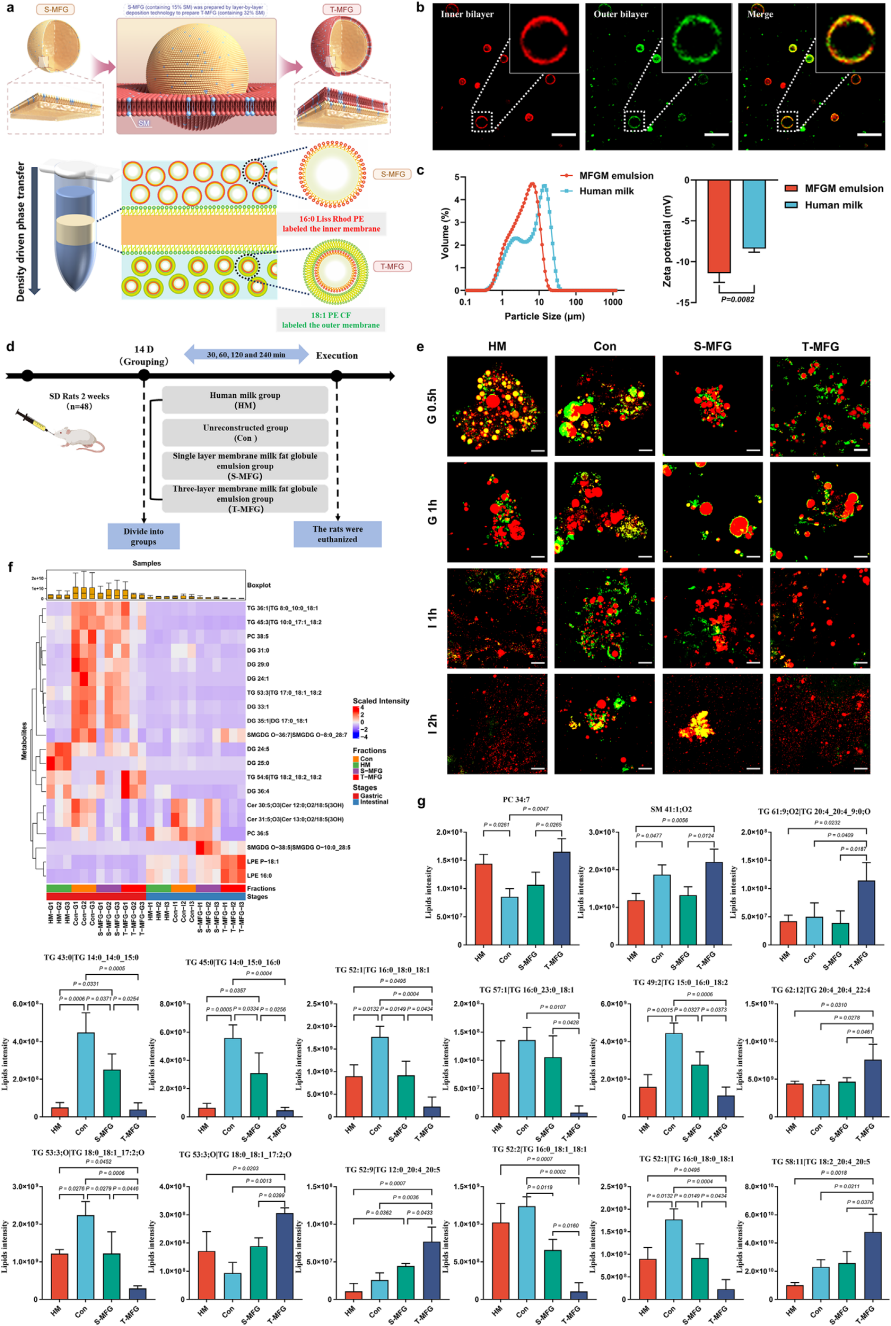
Reconstruction of tri‐layer milk fat globules (T‐MFG) integrating milk fat globule membrane (MFGM) lipid composition remodeling and membrane structure reconstruction, and their in vivo digestion characteristics. a) Schematic illustration of the T‐MFG preparation strategy, which combines both MFGM phospholipid composition remodeling with tri‐layer membrane structure reconstruction. Specifically, cow milk‐derived MFGM phospholipids were supplemented with sphingomyelin to match the phospholipid profile of human milk (HM), followed by layer‐by‐layer deposition to reconstitute the characteristic tri‐layer membrane structure of native human milk fat globules. b) Confocal laser scanning microscopy (CLSM) image showing the glyceride core surrounded by a monolayer of MFGM (inner layer, red fluorescence) and an additional bilayer forming the outer membrane (green fluorescence), resulting in a tri‐layer structure. Scale bar = 10 µm. c) Comparison of particle size and zeta potential between T‐MFG and HM. d) Schematic of the in vivo experimental design used to evaluate gastrointestinal digestion in neonatal rat pups. Treatment groups included HM, control (Con), single‐layer MFG (S‐MFG), and T‐MFG. e) CLSM images in MFG during in vivo digestion. (Green fluorescence indicates phospholipids, red indicates glycerides). Both T‐MFG and HM maintained smaller aggregate sizes and better structural integrity during the gastric and intestinal phases. Scale bar = 20 µm. f) Heatmap analysis of common significantly different lipid species among HM, Con, S‐MFG, and T‐MFG groups during gastric and intestinal digestion. g) Quantitative analysis of significantly altered lipid species detected in the serum of rat pups across different treatment groups.

In this study, cow milk‐derived MFGM was used as a raw material. Its MFGM phospholipid composition was modified to reconstruct a tri‐layer milk fat globule (T‐MFG) exhibiting structural characteristics similar to those of HM, using layer‐by‐layer deposition technology (**Figure** **2**a). Confocal laser scanning microscopy (CLSM) characterization confirmed the information of a glyceride core surrounded by a monolayer of MFGM (inner layer, red fluorescence) and a tri‐layer structure with two additional MFGM layers forming the outer layer (green fluorescence) (Figure 2b). The humanized T‐MFG exhibited a unimodal particle size distribution, in contrast to the bimodal distribution observed in natural HM (Figure 2c). The particle size of the T‐MFG was 5.594 µm, smaller than the 9.943 µm observed in human MFG. Additionally, the zeta potential of the reconstructed T‐MFG was slightly lower than that of HM (Figure 2c).

To further assess the digestive properties of the humanized MFG, the study compared the in vitro gastrointestinal digestion characteristics of a non‐reconstructed control (Con) sample, HM, single‐layer milk fat globules (S‐MFG), and the reconstructed T‐MFG under simulated infant gastrointestinal digestion (Figure S2, Supporting Information). All groups exhibited significant structural transformations during simulated digestion. Initially, triacylglycerols (TAGs) in HM displayed a spherical configuration, with phospholipids arranged in a ring‐like structure at the surface (Figure S2a, Supporting Information). The S‐MFG and T‐MFG exhibited similar TAG and phospholipid structural arrangements, while the Con group displayed larger TAG aggregates, consistent with particle size distribution results (Figure S2b, Supporting Information). After 1 h of gastric digestion, the phospholipid layer surrounding the fat globules played a key role in modulating the progression of digestion within the gastrointestinal tract. At the end of gastric digestion, the phospholipid‐encapsulated structures remained relatively intact in both HM and T‐MFG groups, suggesting that the multilayered phospholipid coating helped delay TAG hydrolysis by gastric lipases. During the early phase of intestinal digestion (I 1h), large aggregates formed during the gastric phase gradually decreased as digestion advanced. By 2 h into intestinal digestion (I 2h), most aggregates had dispersed, indicating significant hydrolysis of TAGs and phospholipids in all groups. Overall, the T‐MFG developed in this study demonstrated digestion behavior that more closely resembled that of HM fat when compared to both the Con and S‐MFG groups. Additionally, T‐MFG effectively minimized flocculation and aggregation throughout digestion, highlighting its potential as a model for studying HM fat digestion and guiding the design of infant nutritional formulations that more accurately resemble the digestive behavior of natural milk fat.

### 2.3 In Vivo Digestion, Lipidomics, and Metabolomics Analysis of Humanized Milk Fat Globules: Insights into Lipid Absorption and Neurodevelopment

This study provides a deeper understanding of the digestion characteristics of humanized MFG by analyzing in vivo gastrointestinal digestion patterns. A rat pup model was employed to evaluate the digestion and metabolism of humanized MFG, focusing on how the structural properties of different MFG samples influence digestion under physiological conditions (Figure 2d). After 0.5 h of in vivo gastric digestion, significant differences in fat globule structural integrity were observed across the groups. The HM and T‐MFG groups maintained intact fat globules, indicating that their structural stability closely resembled that of HM. In contrast, the S‐MFG group exhibited complete disintegration of the fat globules, indicating decreased stability under gastric conditions (Figure 2e). These findings suggested that the multilayered structure of T‐MFG provided enhanced protection for the lipid core against gastric enzyme breakdown, thereby preserving a structure that more closely resembles human MFG. Further examination after in vivo intestinal digestion showed that the Con samples retained significant levels of undigested TAGs and phospholipids (Figure 2e). In contrast, the HM and T‐MFG groups exhibited a more extensive breakdown of TAGs and phospholipids.

The digestion and absorption mechanisms of the humanized MFG were further investigated using lipidomic and metabolomic analysis, aiming to reveal pathways associated with lipid utilization and cognitive development. The lipid and metabolite composition of gastric contents, intestinal contents, and serum samples were analyzed to gain a deeper understanding of the digestion process and its potential effect on neural development.

#### 2.3.1 Gastric Phase: Lipid and Metabolite Composition

The effect of humanized MFG on the lipid composition of gastric contents was investigated using lipidomics analysis. The results from the partial least squares discriminant analysis (PLS‐DA) showed that the HM and T‐MFG samples clustered on the left side of principal component 1, indicating that the lipid composition of the gastric contents in the T‐MFG group was comparable to that of HM, whereas the S‐MFG and Con groups were positioned on the right side of principal component 1 (Figure S3a, Supporting Information). Furthermore, correlation analysis was performed to assess the similarity in lipid composition among the different MFG samples (Figure S3b, Supporting Information). The results indicated that the correlation coefficients between T‐MFG and HM were higher than those between Con and HM, with the mean correlation coefficient of T‐MFG and HM (0.87) exceeding that of S‐MFG and HM (0.81). Phospholipid class analysis revealed that, during the gastric digestion phase, the levels of phosphatidylcholine (PC) and phosphatidylserine (PS) in the Con group differed significantly from those in HM, whereas the phospholipid classes in both S‐MFG and T‐MFG did not exhibit significant differences when compared to HM (Figure S3c–g, Supporting Information). To provide a comprehensive and intuitive representation of the interactions among the samples, heatmap analysis was performed for the significantly different lipid species (Figure S3h, Supporting Information). The results demonstrated differences in lipid expression among the MFG samples during gastric digestion, indicating that the MFG structure had a significant impact on lipid digestion. Notably, the overall trend in lipid species abundance in T‐MFG was comparable to that in HM. The significantly different lipid species primarily included diglyceride (DG, 55, 23.01%), ceramides (Cer, 20, 8.37%), SM (20, 8.37%), and triglyceride (TG, 20, 8.37%) (Figure S3i, Supporting Information). Further analysis of the KEGG metabolic pathways associated with the significantly different lipid species revealed their roles in glycerophospholipid metabolism, as well as in pathways related to fat and vitamin digestion and absorption (Figure S3j, Supporting Information). A comparative analysis was conducted to examine the effects of different MFG structures on lipid digestion and absorption during the gastric phase, focusing on lipid species differences among the groups. A comparison of lipid species abundances in HM, T‐MFG, S‐MFG, and Con revealed that lipid species such as SM 39:7;O2, LPA 21:3, LPE 18:1, and PI 35:3;O|PI 18:1_17:2;O were significantly lower in the HM and T‐MFG groups than in the Con and S‐MFG groups (Figure S3k, Supporting Information).

Metabolomic analysis of gastric contents was conducted to assess the impact of humanized MFG. The principal component analysis (PCA) analysis revealed that HM and T‐MFG clustered closely together on the left side of principal component 1 (Figure S4a, Supporting Information), while S‐MFG and Con were positioned on the right, indicating a high degree of similarity in the metabolomic profiles of HM and T‐MFG during the gastric phase. A total of 367 significant metabolites were identified among the differential metabolites in gastric contents, with the most abundant classes being fatty acyls (51, 13.9%), carboxylic acids and derivatives (47, 12.81%), prenol lipids (38, 10.35%), and steroids and steroid derivatives (35, 9.54%) (Figure S4b, Supporting Information). Hierarchical clustering of the top 50 significant differential metabolites based on the variable importance in the projection (VIP) scores revealed that the metabolic composition of S‐MFG and Con was comparable, whereas HM and T‐MFG clustered separately (Figure S4c, Supporting Information). Pathway enrichment analysis of these metabolites demonstrated that the metabolic changes observed during the gastric phase were primarily associated with lipid metabolism and digestion‐associated pathways (Figure S4d, Supporting Information). In summary, the lipid and metabolite profiles of T‐MFG during the gastric phase closely resembled those of HM.

#### 2.3.2 Intestinal Phase: Digestion and Lipid Stability

The effect of humanized MFG on the lipid composition of intestinal contents was investigated using lipidomics analysis. PLS‐DA of the lipidomic data revealed distinct compositional differences among the MFG samples during the intestinal digestion phase (Figure S5a, Supporting Information). Specifically, HM and T‐MFG clustered on the lower side of principal component 2, indicating that the lipid composition in the T‐MFG group closely resembled that of HM. In contrast, S‐MFG and Con samples clustered on the upper side. Correlation analysis was subsequently performed to assess the similarity in lipid composition among the different MFG samples (Figure S5b, Supporting Information). The average correlation coefficient between T‐MFG and HM was 0.76, lower than that between S‐MFG and HM (0.79). All correlation coefficients were below 0.8, indicating a moderate positive correlation between the MFG groups and HM. Phospholipid class analysis revealed that SM levels in the T‐MFG group were significantly higher than those in HM during the intestinal digestion phase (Figure S5c–g, Supporting Information). Heatmap analysis of differential lipids indicated that the lipid molecular composition of T‐MFG remained highly comparable to that of HM during intestinal digestion (Figure S5h, Supporting Information). The major differential lipid classes included TG (74, 26.24%), Cer (36, 12.77%), DG (31, 10.99%), and PC (22, 7.8%), with TG comprising a significantly higher proportion compared to the gastric phase (Figure S5i, Supporting Information). KEGG pathway enrichment analysis showed that the differentially abundant lipid species were primarily involved in glycerophospholipid metabolism, as well as lipid digestion and absorption pathways, consistent with the core metabolic activities observed in the gastric phase (Figure S5j, Supporting Information). Comparison of lipid species among HM, T‐MFG, S‐MFG, and Con revealed that, among the 14 significantly different lipids, 9 Cer species and 2 PC species were present at significantly lower levels in T‐MFG than in S‐MFG (Figure S5k, Supporting Information).

The effect of T‐MFG on metabolite composition during the intestinal digestion phase was investigated using metabolomics analysis. PCA revealed that HM and T‐MFG clustered along the lower side of principal component 2, whereas S‐MFG and Con were distributed along the upper side (Figure S6a, Supporting Information). A total of 93 significantly different metabolites were identified, with prenol lipids (13, 14.29%) and carboxylic acids and derivatives (11, 12.09%) representing the predominant classes (Figure S6b, Supporting Information). Heatmap clustering analysis of the top 50 significant metabolites, ranked by VIP scores, indicated that S‐MFG and Con exhibited highly comparable metabolite compositions, whereas HM and T‐MFG formed distinct clusters (Figure S6c, Supporting Information). Pathway enrichment analysis of the differential metabolites demonstrated that the significant metabolic changes during the intestinal phase were associated with signaling pathways involved in digestive processes (Figure S6d, Supporting Information).

To further investigate lipid composition changes during in vivo gastrointestinal digestion, 20 significantly different lipid species common to gastrointestinal digestion were identified across the HM, Con, S‐MFG, and T‐MFG groups. These lipid species were subsequently analyzed using hierarchical clustering (Figure 2f). The overall variation trend of the 20 lipid species observed during the gastric digestion phase indicated that T‐MFG closely resembled HM, whereas S‐MFG showed a trend comparable to Con.

#### 2.3.3 Serum Composition: Circulatory Lipid and Metabolite Distribution

The effect of humanized MFG on serum lipid composition was investigated using lipidomics analysis. PLS‐DA revealed distinct distribution patterns of serum lipids across treatment groups (Figure S7a, Supporting Information). HM and T‐MFG samples clustered on the left side of principal component 1, while the S‐MFG group was positioned near the origin, and the Con group clustered on the right. This distribution indicated that the serum lipid profile of T‐MFG closely resembled that of HM, whereas S‐MFG exhibited an intermediate lipid profile between T‐MFG and Con. Correlation analysis was performed to evaluate the effects of different MFG treatments on serum lipid composition (Figure S7b, Supporting Information). The mean correlation coefficients between HM and the T‐MFG, S‐MFG, and Con groups were all higher than 0.8, indicating a strong positive correlation between the lipid profiles of all treatment groups and HM. Analysis of phospholipid classes in serum (Figure S7c–g, Supporting Information) revealed no significant differences in phospholipid content among the groups. A differential lipid heatmap analysis was conducted to assess the effects of different MFG treatments on serum lipid composition (Figure S7h, Supporting Information). The results demonstrated a strong similarity in serum lipid profiles between HM and both the S‐MFG and T‐MFG groups. Despite these similarities, 226 significantly different lipid species were identified across the groups. Further categorization of these differential lipids indicated that the predominant classes included TG (84, 39.07%), PC (22, 11.16%), oxidized triacylglycerols (OxTG, 16, 6.98%), and phosphatidylinositol (PI, 12, 5.58%) (Figure S7i, Supporting Information). To investigate the functional implications of these lipids, KEGG pathway enrichment analysis was performed (Figure S7j, Supporting Information). The results indicated that the differential lipids were predominantly associated with lipid metabolism pathways, including glycerophospholipid metabolism, and neurobiological pathways, such as retrograde endocannabinoid signaling. Among the 15 significantly different lipid species, 13 were identified as TGs (Figure 2g). Serum TG levels were consistently lower in T‐MFG and HM groups, whereas S‐MFG and Con groups exhibited higher concentrations. Additionally, PC 34:7 and SM 41:1;O2 levels were significantly higher in the T‐MFG group compared to the S‐MFG group.

This study further investigated the impact of T‐MFG on serum metabolite composition through metabolomic analysis. PCA results indicated no significant differences in metabolite composition among the four groups, with all samples clustering near the origin (Figure S8a, Supporting Information). A total of 46 significant metabolites were identified, with the highest proportion belonging to fatty acyl compounds (9, 19.57%), followed by glycerophospholipids (8, 17.39%) and carboxylic acid derivatives (4, 8.7%) (Figure S8b, Supporting Information). Hierarchical clustering of the top 50 metabolites, based on VIP scores, revealed that the metabolite composition of the S‐MFG and Con groups was highly comparable, where these groups were distinctly separated from the HM and T‐MFG groups (Figure S8c, Supporting Information). KEGG pathway enrichment analysis of differential metabolites showed that these metabolites were primarily associated with lipid metabolism and signal transduction pathways (Figure S8d, Supporting Information). In summary, while differential metabolites were identified among the groups, metabolomic analysis revealed that the overall serum metabolite composition remained generally consistent.

### 2.4 Humanized MFG Promotes Reflex Development and Hippocampal Structure in Rat Pups

**Figure 3:**
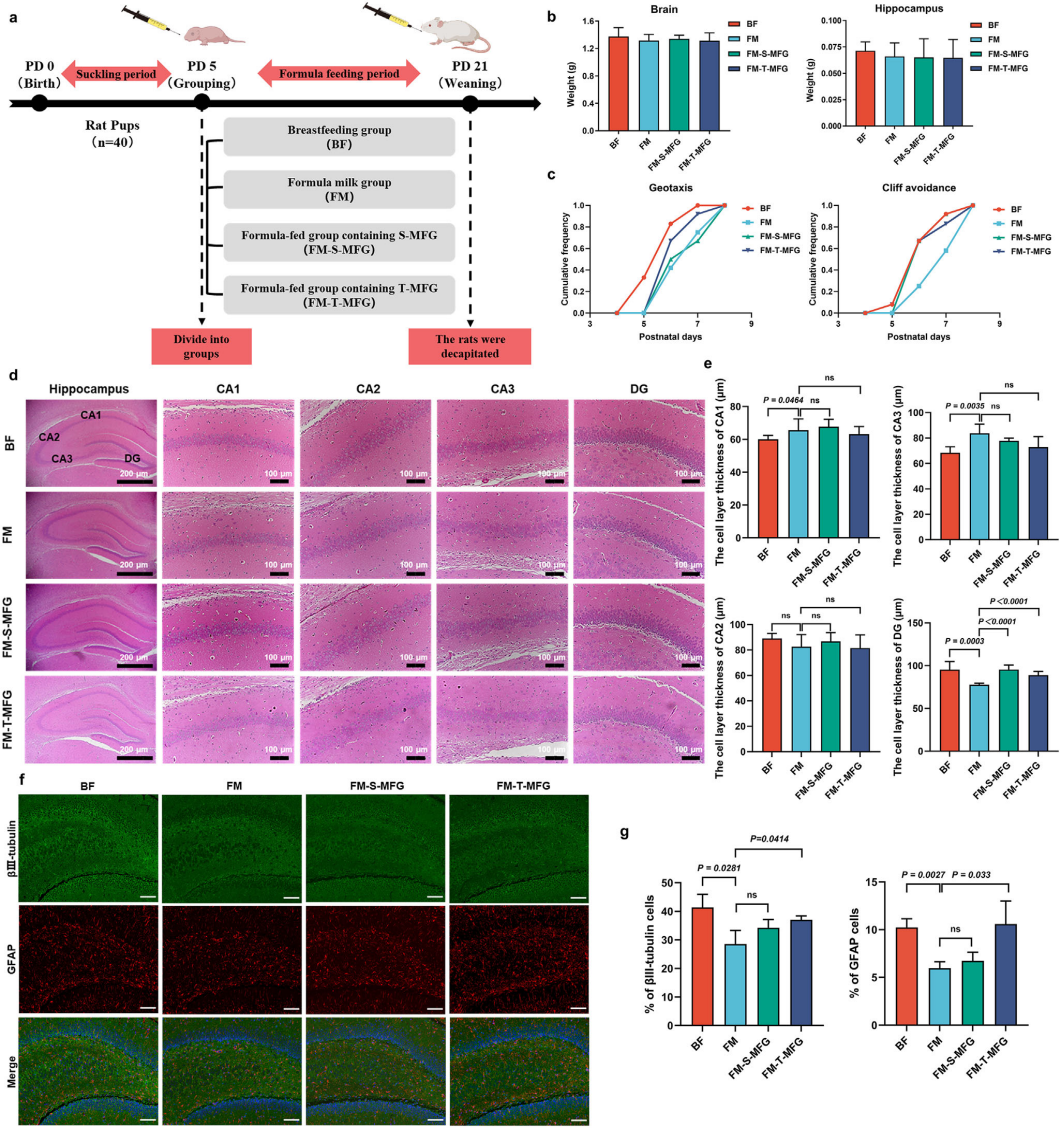
Effects of humanized milk fat globules (MFG) on cognitive behavior and hippocampal histopathology in rat pups. a) Experimental design and group allocation for rat pups fed different types of formula milk. Neonatal rats were randomly assigned to four groups: breastfed (BF), formula milk (FM), formula supplemented with single‐layer MFG (FM‐S‐MFG), and formula supplemented with tri‐layer MFG (FM‐T‐MFG) (n=10). b) Brain and hippocampal weights of rat pups across the different formula milk groups (n=10). c) Behavioral assessments of geotaxis and cliff avoidance in rat pups fed humanized formula milk compared to the BF group (n=10). d) Representative hematoxylin and eosin (H&E)‐stained hippocampal sections. The BF and FM‐T‐MFG groups exhibited well‐preserved hippocampal structures, while the FM group showed a thinner neuronal layer and decreased cell density in the dentate gyrus (n=3). e) Quantification of hippocampal neuronal layer thickness revealed that supplementation with FM‐S‐MFG or FM‐T‐MFG partially restored hippocampal architecture compared to the FM group (n=3). f) Representative immunofluorescence images of βIII‐tubulin (neuronal marker, green fluorescence) and glial fibrillary acidic protein (GFAP, astrocyte marker, red fluorescence) in the hippocampus. Scale bar = 200 µm (n=3). g) Quantitative analysis of immunofluorescence intensity showed significantly increased levels of βIII‐tubulin and GFAP in the FM‐T‐MFG group compared to the FM group, with no significant difference from the BF group, suggesting enhanced neurogenesis and glial support (n=3).

A formula‐fed rat model was employed to investigate the effects of humanized MFG on cognitive development in rat pups (**Figure** **3**a). Compared to the formula milk (FM), rat pups in the FM‐S‐MFG (formula supplemented with single‐layer MFG) and FM‐T‐MFG (formula supplemented with tri‐layer MFG) did not show a significant increase in brain hippocampal weight (Figure 3b). Behavioral assessments revealed no significant differences among the four treatment groups in eyelid twitching, grip strength, tooth eruption, or eyes opening maturation days (Figure S9, Supporting Information). However, a notable finding was that the FM‐S‐MFG and FM‐T‐MFG groups exhibited a significantly earlier onset of the cliff avoidance reflex than the FM group. Notably, the timing of reflex onset in these two groups was comparable to that of the breastfed (BF) group (Figure 3c). Additionally, geotactic reflex onset was significantly delayed in the FM and FM‐S‐MFG groups compared to the BF group, while the FM‐T‐MFG group showed no significant difference from the BF group.

Neuronal morphological changes in rat pup brain tissues were conducted using hematoxylin and eosin (H&E) staining. The BF group displayed no pathological changes (Figure 3d), and the hippocampal tissue structure appeared intact, with neurons exhibiting normal rounded or pyramidal morphology. In contrast, the FM group showed a reduced neuronal layer thickness and a decreased neuronal count in the dentate gyrus region compared to the other treatment groups (Figure 3e). However, supplementation with FM‐S‐MFG and FM‐T‐MFG significantly restored the thickness of the cell layer in the dentate gyrus region, increased neuronal density, and promoted a more organized cellular arrangement. Additionally, supplementation with humanized MFG enhanced the proportion of neurons in the dentate gyrus region (Figure 3f). The number of βIII‐tubulin‐ and GFAP‐positive cells in the FM‐T‐MFG group was significantly higher than in the FM group, with no significant difference observed compared to the BF group (Figure 3g).

### 2.5 Humanized MFG Alters Serum Metabolite Profile and Enhances Cognitive‐Related Pathways in Rat Pups

**Figure 4:**
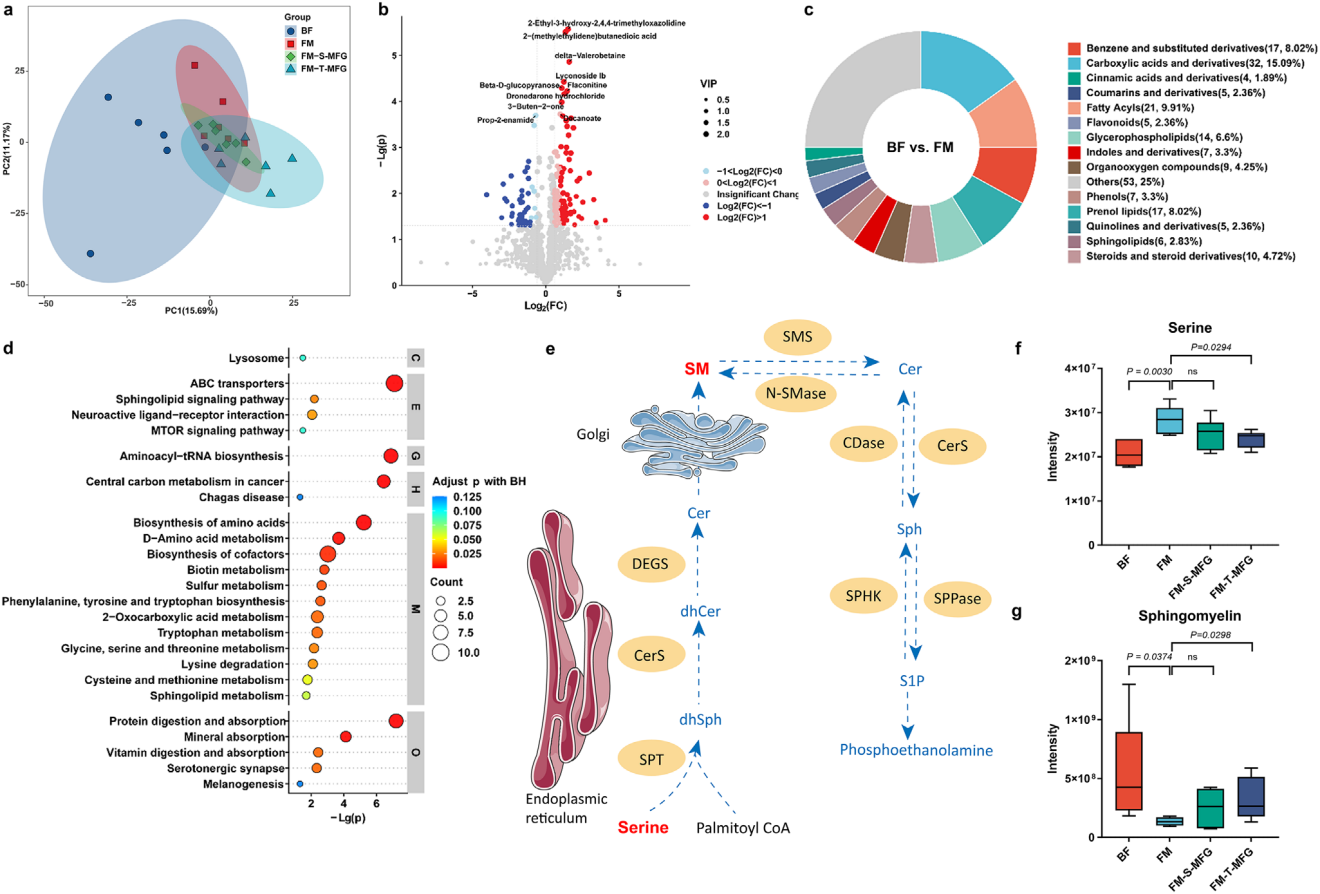
Effects of humanized milk fat globules (MFG) on serum metabolite composition in rat pups. a) Principal component analysis (PCA) of serum metabolites revealed distinct metabolic profiles among treatment groups. The breastfed (BF) group exhibited a clearly separated profile, while the formula‐fed groups, including formula milk (FM), formula supplemented with single‐layer MFG (FM‐S‐MFG), and formula supplemented with tri‐layer MFG (FM‐T‐MFG), clustered more closely together. b) Volcano plot showing significantly different serum metabolites between the BF and FM groups. Red dots represent upregulated metabolites; blue dots represent downregulated metabolites. c) Classification of 217 significantly differential metabolites, primarily including carboxylic acids and derivatives, as well as fatty acyls. d) KEGG pathway enrichment analysis showing cognitive‐related metabolic pathways affected by formula feeding, including neuroactive ligand‐receptor interaction, synaptic metabolism, and sphingolipid signaling pathways. e) Schematic illustration of the sphingolipid signaling pathway. f) Comparison of serum serine levels across different formula‐fed groups. g) Comparison of serum SM levels across different formula‐fed groups (n=6).

Metabolomic analysis was performed across different feeding groups to assess the impact of humanized MFG on serum metabolite composition in rat pups, revealing significant metabolite differences. As shown in **Figure** **4**a, the serum metabolite profiles of all formula‐fed groups differed significantly from those of the breastfed group, while no significant differences were observed among the formula‐fed groups. Volcano plot analysis identified 217 significantly different metabolites between the BF and FM groups (Figure 4b), primarily comprising carboxylic acids and derivatives, as well as fatty acyls (Figure 4c). Many of these metabolites are involved in cognitive‐related pathways, including neuroactive ligand‐receptor interaction, synaptic energy metabolism, sphingolipid signaling, and melanin biosynthesis (Figure 4d). These pathways play key roles in neuronal communication, energy supply, and cell membrane integrity, all essential for cognitive function. Further comparison of metabolites within the sphingolipid signaling pathway revealed that the FM‐T‐MFG group exhibited a distinct profile characterized by significantly elevated serum serine and SM levels (Figure 4e–g). This selective upregulation of cognition‐related metabolites suggested that supplementation with humanized MFG may provide metabolic benefits, potentially enhancing cognitive function through an enriched serum metabolite profile.

### 2.6 Humanized MFG Modulates Hippocampal Lipid and Protein Profiles, Enhancing Cognitive Pathways in Rat Pups

**Figure 5:**
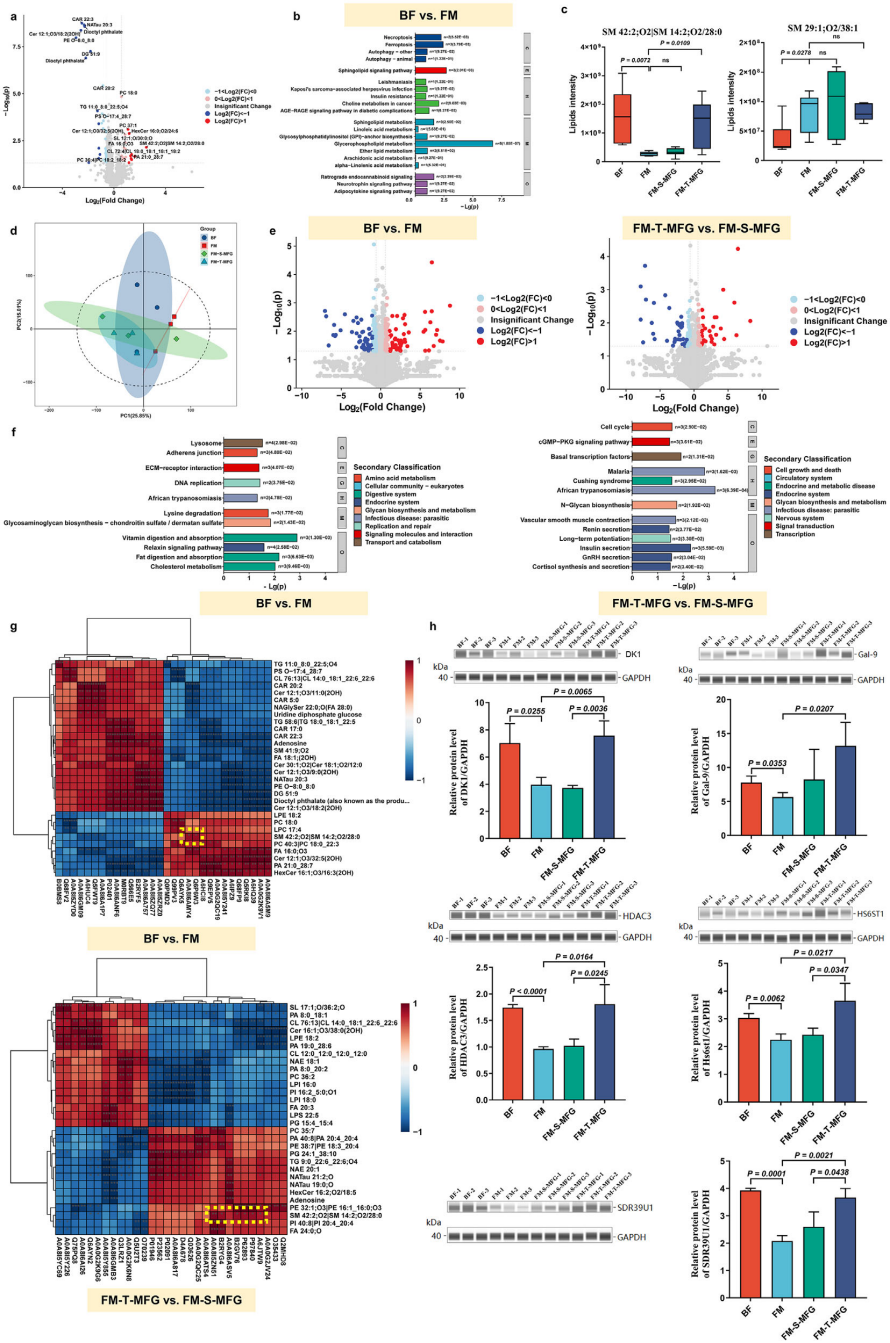
Regulation of hippocampal lipid and protein composition by humanized milk fat globules (MFG) in formula‐fed rat pups. a) Volcano plot showing significantly different hippocampal lipid species between the breastfed (BF) and formula milk (FM) groups. Red dots indicate upregulated lipid species, and blue dots indicate downregulated lipid species. b) KEGG pathway enrichment analysis of significantly altered hippocampal lipids between the BF and FM groups, highlighting cognition‐related pathways such as retrograde endocannabinoid signaling and neurotrophin signaling. c) Relative abundance of selected significantly altered lipid species across the BF, FM, FM‐S‐MFG (formula supplemented with single‐layer MFG), and FM‐T‐MFG (formula supplemented with tri‐layer MFG) groups. d) Principal component analysis (PCA) of global hippocampal protein expression, showing overall similarities and differences among groups. e) Volcano plot highlighting differentially expressed hippocampal proteins. Blue dots indicate significantly downregulated proteins; red dots indicate significantly upregulated proteins. f) KEGG pathway enrichment analysis of significantly altered hippocampal proteins. Proteins differentially expressed between the FM and BF groups were primarily associated with extracellular matrix (ECM)–receptor interaction pathways. In contrast, proteins differing between FM‐S‐MFG and FM‐T‐MFG groups were enriched in pathways related to cGMP–PKG signaling and long‐term potentiation. g) Correlation analysis between significantly changed hippocampal lipids and proteins. Notably, SM 42:2;O2 exhibited strong positive correlations with several key proteins. h) Representative immunoblot images and quantitative analysis of dolichol kinase (DK1), Galectin‐9 (Gal‐9), histone deacetylase 3 (HDAC3), heparan‐sulfate 6‐O‐sulfotransferase 1 (HS6ST1), and short‐chain dehydrogenase/reductase family 39U member 1 (SDR39U1) in the hippocampus of rat pups treated with humanized MFG. Glyceraldehyde‐3‐phosphate dehydrogenase (GAPDH) served as the loading control (n=3).

Lipidomic analysis was performed to assess the hippocampal lipid composition across different feeding groups and investigate the impact of humanized MFG on cognitive development. A total of 64 lipid classes and 1,341 lipid species were detected in the hippocampus. Statistical comparisons identified 56 significantly altered lipid species between the FM and BF groups. These differential lipids were closely associated with cognition‐related pathways, including retrograde endocannabinoid and neurotrophin signaling (**Figure** **5**a,b). Further analysis of phospholipid profiles (Figure 5c) showed that supplementation with humanized MFG significantly increased hippocampal SM levels, particularly SM 42:2; O2.

In addition, proteomic analysis was employed to investigate the regulatory characteristics of different feeding regimens on hippocampal protein expression. PCA results indicated no significant separation in the overall protein expression profiles among the groups (Figure 5d), suggesting that humanized MFG had minimal impact on the overall proteomic differences in the hippocampus. To investigate differences in hippocampal protein composition across feeding groups, proteins with significant differential expression were identified based on the criteria of *P* < 0.05 and FC ≥ 1.5 or FC ≤ 0.667 (Figure 5e). Between the FM and BF groups, 200 significantly different proteins were identified, with 101 upregulated and 99 downregulated. In comparison, 119 differentially expressed proteins were identified between the FM‐T‐MFG and FM‐S‐MFG groups, including 55 upregulated and 64 downregulated proteins. KEGG pathway enrichment analysis was performed to elucidate the functional implications of humanized MFG on hippocampal protein expression (Figure 5f). The differentially expressed proteins between the FM and BF groups were primarily enriched in the extracellular matrix (ECM)‐receptor interaction pathway. In contrast, proteins differentiating the FM‐T‐MFG and FM‐S‐MFG groups were significantly enriched in the cGMP‐PKG signaling pathway and long‐term potentiation, which are associated with signal transduction and the neural function.

Correlation analysis was conducted to investigate the relationship between hippocampal lipid and protein variations (Figure 5g). The results revealed a significant positive correlation between SM 42:2;O2 and several proteins, including dolichol kinase (DK1), galectin‐9 (Gal‐9), histone deacetylase 3 (HDAC3), heparan‐sulfate 6‐O‐sulfotransferase 1 (HS6ST1), and short‐chain dehydrogenase/reductase family 39U member 1 (SDR39U1). The expression of these target proteins was further validated using JSEE (Figure 5h). DK1, HDAC3, HS6ST1, and SDR39U1 were significantly upregulated in the FM‐T‐MFG group compared to the FM‐S‐MFG and FM groups, with no significant difference from the BF group. Additionally, Gal‐9 expression was significantly higher in the FM‐T‐MFG group than in the FM group, while no significant difference was observed between the FM‐T‐MFG and FM‐S‐MFG groups.

### 2.7 Humanized MFG Modulates Gut Microbiota Composition in Formula‐Fed Rat Pups, Potentially Supporting Cognitive Development

**Figure 6:**
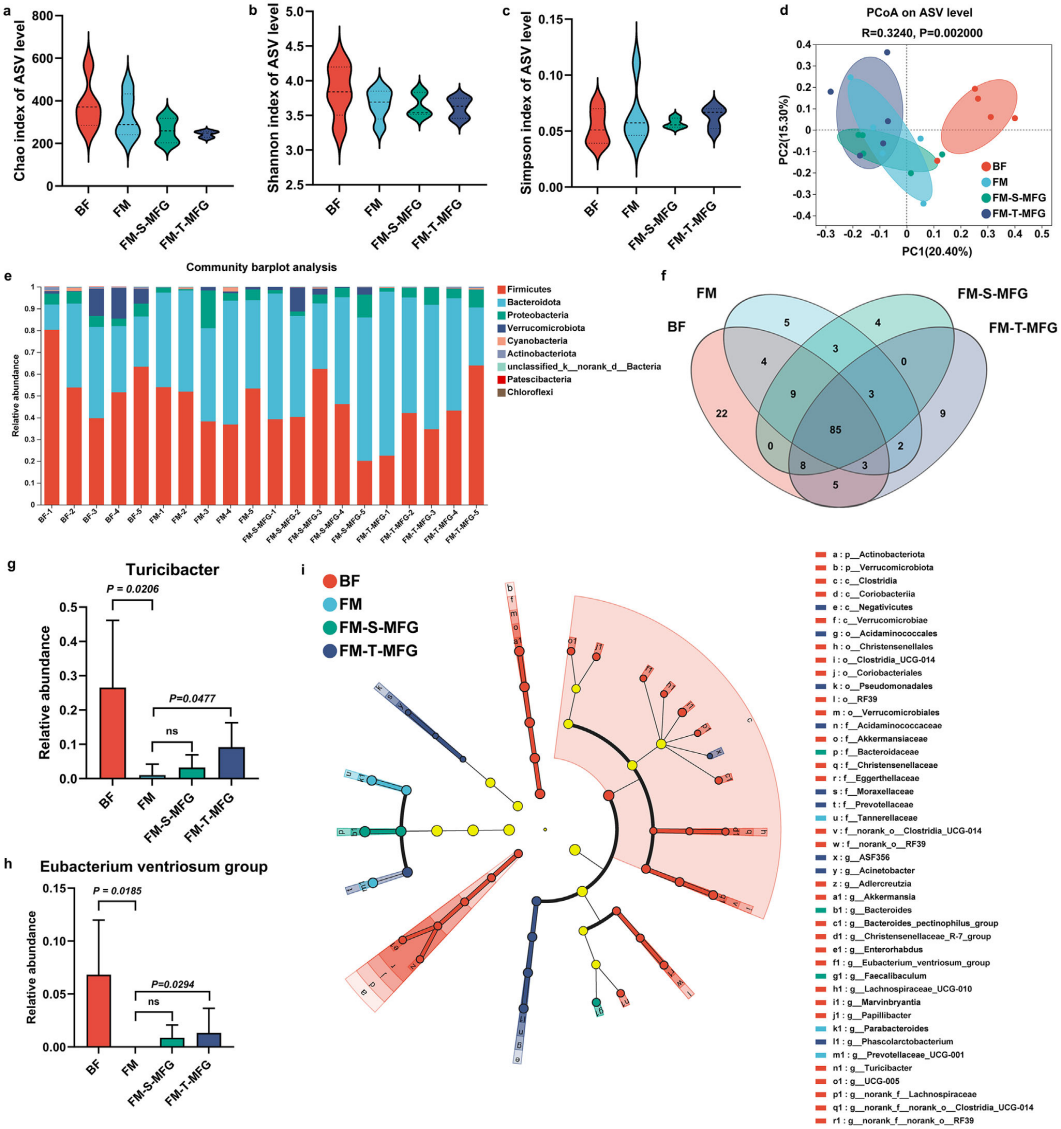
Impact of humanized milk fat globules (MFG) on gut microbiota composition in rat pups. a–c) Alpha diversity metrics of the gut microbiota based on 16S rRNA gene sequencing of colonic contents from postnatal day 21 rat pups. The Chao1 index (a), Shannon index (b), and Simpson index (c) were used to assess microbial species richness and diversity. d) Principal coordinate analysis (PCoA) based on Bray‐Curtis distances at the amplicon sequence variant (ASV) level, showing distinct clustering between the breastfed (BF) and formula‐fed groups, indicating significant differences in microbial community composition (beta diversity). e) Bar plot showing the relative abundance of gut microbiota at the phylum level. Formula‐fed pups exhibited decreased Firmicutes and increased Bacteroidota compared to the BF group. f) Venn diagram showing the number of unique and shared bacterial genera among the four groups: BF, formula milk (FM), formula supplemented with single‐layer MFG (FM‐S‐MFG), and formula supplemented with tri‐layer MFG (FM‐T‐MFG). (g‐h) Relative abundances of two cognition‐associated bacterial genera: *Turicibacter* (g) and *Eubacterium ventriosum group* (h). These were significantly increased in the FM‐T‐MFG group compared to the FM and FM‐S‐MFG groups. i) Linear discriminant analysis effect size (LEfSe) identified taxa with significantly different relative abundances among groups. Notably, the BF group was enriched in *Clostridia* and *Akkermansia*, both of which are associated with gut health and neurodevelopment (n=5).

Emerging evidence suggests that the gut microbiota plays a critical role in shaping cognitive development in infants. Given the observed cognitive benefits of humanized MFG, this study investigated whether MFG supplementation also influenced gut microbiota composition in formula‐fed rat pups. To address this, 16S rRNA gene sequencing was conducted on colonic contents from four groups: BF, FM, FM‐S‐MFG, and FM‐T‐MFG. Alpha diversity indices, including Chao, Shannon, and Simpson, were analyzed to assess microbial richness and community diversity. As shown in **Figure** **6**a–c, no significant differences were detected in α diversity across the formula‐fed groups compared to the BF group, indicating that humanized MFG did not significantly alter microbial richness or evenness. However, principal coordinate analysis (PCoA) of beta diversity revealed significant compositional differences in gut microbiota among the formula‐fed and BF groups (Figure 6d). At the phylum level, taxonomic analysis indicated a distinct shift in microbial composition in formula‐fed pups, characterized by a significant decrease in the relative abundance of Firmicutes and an increase in Bacteroidota compared to BF pups (Figure 6e). This shift suggests that formula feeding, regardless of MFG supplementation, induced significant changes in gut microbial composition at the phylum level. At the genus level, unique and overlapping microbial taxa were identified across different feeding groups, providing insights into the specific microbial alterations associated with humanized MFG supplementation. The BF group exhibited 22 unique genera, whereas the FM group had only 5. Notably, the FM‐S‐MFG and FM‐T‐MFG groups displayed 4 and 9 unique genera, respectively, suggesting that humanized MFG supplementation contributed to compositional changes in gut microbiota by introducing distinct microbial taxa (Figure 6f). Further analysis revealed that the FM‐T‐MFG supplementation increased relative abundances of *Turicibacter* and the *Eubacterium ventriosum* group compared to the FM‐S‐MFG group (Figure 6g,h). This increase in these beneficial bacterial taxa highlights the potential role of MFG supplementation in selectively enhancing gut microbiota profiles in a way that might support improved gut health and cognitive outcomes. To further elucidate taxonomic differences, linear discriminant analysis effect size (LEfSe) was used to identify differentially abundant taxa among the groups. As shown in Figure 6i, the BF group showed significant enrichment of the class *Clostridia* and genus *Akkermansia*, both of which have been associated with beneficial gut functions.

## 3 Discussion

Emerging trends and challenges in fat modification for next‐generation infant formula emphasize the importance of mimicking the phospholipid composition of the MFGM and the structure of MFG to improve lipid digestion and metabolism in infants while also investigating their potential effects on cognitive development.^[^ ^10^ ^]^ Our previous research highlighted significant differences between cow milk and human MFGM, particularly in SM content, ≈32% in human MFGM compared to only 15% in cow MFGM.^[^ ^15^ ^]^ Although multiple preclinical and clinical studies have demonstrated that cow‐derived MFGM supplementation can enhance cognitive function in both infants and rodent models, its efficacy may be limited by inherent compositional differences and processing‐induced disruptions to membrane integrity.^[^ ^16^, ^17^ ^]^ To address these limitations, the present study successfully constructed a humanized MFG (T‐MFG) using a layer‐by‐layer deposition technique that combined both “MFGM lipid composition remodeling” and “tri‐layer membrane structure reconstruction.” This dual strategy sets our approach apart from conventional cow MFGM supplementation, which generally increases total phospholipid content without replicating the native phospholipid composition or trilayer structure of human MFG. The effects of humanized MFG on various biological and cognitive markers in formula‐fed rat pups were evaluated to address developmental differences between formula‐fed and breastfed pups. Our findings indicated that supplementation with humanized MFG influences multiple outcomes, including neurobehavioral development, hippocampal morphology, gut microbiota composition, serum metabolites, lipidomic profiles, and proteomic signatures. Notably, the MFG‐supplemented groups exhibited changes that more closely resembled those observed in the breastfed groups. These multidimensional improvements suggest that humanized MFG may support neurodevelopment and cognitive function in formula‐fed pups.

The investigation began with in vitro experiments focusing on the biological effects of humanized MFGM phospholipid composition on NPC. The investigation began with in vitro experiments focusing on the biological effects of humanized MFGM phospholipid composition on NPC. These experiments demonstrated that the humanized MFGM phospholipid composition enhanced NPC proliferation and differentiation, promoted neurosphere growth, and increased βIII‐tubulin expression. Transcriptomic and proteomic analyses further revealed the upregulation of genes and proteins associated with neuroactive and cognitive pathways, including cholinergic synapse and MAPK signaling. Consistent with previous studies, these findings support the notion that lipid droplet availability affects the metabolism and proliferation of NPC, which generate new neurons that integrate into pre‐existing neuronal networks.^[^ ^18^, ^19^ ^]^ Furthermore, we successfully reconstructed a humanized MFG with a tri‐layer membrane structure, incorporating both the phospholipid composition and fat globule structure to mimic the structural and interfacial characteristics of natural breast milk. The unique changes in the interfacial composition and the structure of MFG play a crucial role in regulating lipid digestion.^[^ ^11^, ^20^, ^21^ ^]^ Differences in the physical structure and chemical composition of lipids between infant formula and breast milk can impact lipid digestion, potentially contributing to metabolic disorders during later stages of growth.^[^ ^22^, ^23^ ^]^ Lipidomic and metabolomic analyses conducted across the gastric, intestinal, and serum phases demonstrated that the reconstructed humanized MFG (T‐MFG) closely resembles HM in supporting lipid metabolism, cognitive‐related pathways, and digestive stability. T‐MFG exhibited structural and metabolic similarities to HM, maintaining a stable lipid composition that enhances lipid bioavailability and supports infant brain development. Notably, T‐MFG replicated key metabolic pathways found in HM, including glycerophospholipid metabolism and retrograde endocannabinoid signaling, both associated with neurodevelopment and cognitive function.^[^ ^24^, ^25^, ^26^ ^]^

In behavioral assessments of formula‐fed rat pups, supplementation with humanized MFG positively influenced cognitive reflexes and hippocampal morphology, narrowing the developmental gap typically observed between formula feeding and breastfeeding. The earlier onset of the cliff avoidance reflex in the FM‐T‐MFG group suggests that the enhanced dietary lipid composition may positively influence neural pathways related to cognitive reflexes.^[^ ^27^ ^]^ Additionally, the normalization of geotactic reflex onset times in the FM‐T‐MFG group underscores the potential of humanized MFG in facilitating cognitive maturation. The histopathological analysis further supported these behavioral findings, showing structural improvements in the dentate gyrus region of the hippocampus, which is crucial for hippocampal memory formation, following supplementation with humanized MFG.^[^ ^28^ ^]^ The humanized MFG promoted the restoration of cell layers and increased neuronal density, indicating its potential to normalize hippocampal development in formula‐fed pups. These findings were consistent with the established roles of dietary phospholipids in supporting neurogenesis and neuroplasticity.^[^ ^26^, ^29^, ^30^, ^31^ ^]^

Metabolomic analysis revealed significant changes in serum metabolites following MFG supplementation. These differential metabolites were primarily involved in cognition‐related metabolic pathways, including neuroactive ligand‐receptor interaction and sphingolipid signaling. Notably, the FM‐T‐MFG group showed increased levels of SM in the serum, a lipid critical for brain and nervous system development in infants, regulation of the gut microbiota, and maintenance of the skin barrier.^[^ ^31^ ^]^ In the hippocampal lipidome, supplementation with humanized MFG significantly increased SM levels, particularly SM 42:2; O2, aligning the lipid composition more closely with that observed in breastfed pups. The increased SM levels indicate the formation of a lipid environment conducive to neuronal signaling and cognitive function, as SM is crucial for maintaining membrane fluidity and facilitating synaptic plasticity.^[^ ^32^, ^33^, ^34^ ^]^ The observed differences in cognitive‐related signaling pathways, such as the endocannabinoid and neurotrophin pathways, further emphasize the potential neurodevelopmental benefits of humanized MFG supplementation in promoting optimal lipid profiles. Proteomic profiling further indicated that humanized MFG may support cognitive development through multiple molecular mechanisms. Differentially expressed proteins correlated with increased hippocampal SM 42:2; O2 levels included enzymes and signaling molecules such as DK1, HDAC3, HS6ST1, and SDR39U1, all of which are critical for synaptic function and neurodevelopment. DK1 plays a vital role in dolichol phosphate biosynthesis, a key step in glycosylation processes, and its disruption leads to severe developmental disorders and early infant mortality.^[^ ^35^ ^]^ HDAC3 plays a crucial role in regulating brain functions essential for learning, memory, and other cognitive processes, and its disruption has been linked to cognitive and social impairments.^[^ ^36^, ^37^ ^]^ HS6ST1 is involved in neuronal development by modifying heparan sulfate, which regulates neural migration and intracellular communication, and mutations in this gene can lead to developmental defects, such as infertility and delayed puberty.^[^ ^38^ ^]^ Additionally, SDR39U1, a protein dysregulated in Alzheimer's disease, was significantly upregulated in the brains of formula‐fed pups after FM‐T‐MFG supplementation. These findings suggest that humanized MFG may enhance cognitive development by modulating key molecular pathways involved in neurodevelopment and synaptic function.

Gut microbiota modulation by humanized MFG provides additional insights into its potential neurodevelopmental benefits. Although humanized MFG supplementation did not significantly affect the overall composition or diversity of the gut microbiota in formula‐fed pups, it induced specific genus‐level changes, notably increasing the abundance of beneficial bacteria such as *Turicibacter* and the *Eubacterium ventriosum* group. Previous research has established a strong link between gut microbiota composition and cognitive function, primarily mediated by the microbiota‐gut‐brain axis.^[^ ^39^, ^40^ ^]^ *Turicibacter* plays a critical role in AD by influencing serotonin synthesis and metabolism, thereby modulating the gut‐brain axis.^[^ ^41^, ^42^ ^]^ Although their abundance is reduced in individuals with AD, they are considered a key microbial marker associated with the disease.^[^ ^43^ ^]^ *Eubacterium ventriosum* group, a beneficial butyrate‐producing bacteria, promotes gut health and cognitive function by maintaining gut barrier integrity, modulating immune responses, and enhancing cognitive performance.^[^ ^44^, ^45^, ^46^ ^]^ Moreover, the *Eubacterium ventriosum* group is negatively correlated with inflammatory mediators such as IL‐6 and IL‐8, thereby reducing systemic inflammation and improving gut barrier integrity, which may contribute to the prevention of neurodegeneration associated with AD.^[^ ^46^, ^47^ ^]^ The observed enrichment of microbial taxa linked to cognitive function following humanized MFG supplementation suggests potential neuroprotective effects mediated through gut microbiota modulation, potentially by promoting bacterial species with neuroprotective and anti‐inflammatory properties.

While this study demonstrates the multifaceted benefits of humanized MFG (T‐MFG) supplementation in formula‐fed rat pups, several limitations warrant consideration. First, extrapolating findings from rodent models to human infants must be done with caution. Although rats share key neurodevelopmental and metabolic pathways with humans, notable species‐specific differences exist in lactation biology, gut microbiota composition, brain maturation timelines, and cognitive complexity.^[^ ^48^, ^49^ ^]^ For instance, neurodevelopmental markers such as cliff avoidance and geotaxis, employed in this study, are indirect proxies for more complex cognitive milestones in humans, including social cognition and language acquisition. Second, the controlled laboratory environment and standardized diets used in rodent studies differ significantly from the variable exposures and feeding practices of human infants. Factors such as maternal health, mode of delivery, antibiotic use, and environmental stimuli are known to significantly influence gut microbiota composition and neurodevelopment in humans, potentially altering the response to MFG supplementation.^[^ ^50^, ^51^ ^]^ Third, the duration of the study encompassed only early developmental stages and, therefore, does not permit conclusions regarding long‐term effects on adult health or the potential risk of metabolic or neurodegenerative diseases. Taken together, these limitations suggest that while the rat model provides compelling proof‐of‐concept for the neurodevelopmental and metabolic benefits of humanized MFG, conclusive evidence regarding its efficacy and safety in human infants will require rigorous clinical evaluation. Notably, the mechanistic insights provided by this study offer a robust scientific foundation and specific molecular targets for designing and interpreting future clinical trials. In addition, while the present study primarily focuses on the fundamental biological impact of humanized MFG on early neurodevelopment, the potential economic implications of phospholipid composition remodeling and tri‐layer membrane reconstruction should be addressed in the context of industrial scalability. The added costs associated with precision lipid engineering and structural reconstruction must be balanced against the demonstrated functional benefits and the objective of more closely replicating the composition and structure of human milk. Future cost‐benefit analyses will be crucial to evaluate the commercial feasibility and competitive positioning of humanized MFG as a next‐generation ingredient in the infant formula industry.

In conclusion, this study utilized a multi‐dimensional analysis to investigate the preparation of humanized MFG (T‐MFG) through “MFGM lipid composition remodeling” and “tri‐layer membrane structure reconstruction” while exploring its synergistic regulatory mechanisms in infant lipid metabolism and neurodevelopment. The findings show that humanized MFG, constructed using layer‐by‐layer deposition technology with a three‐layer membrane structure, exhibits excellent digestive and absorption properties. Its dual‐phase regulatory characteristics in the gastric and intestinal phases provide novel insights into lipid metabolism. Furthermore, in addition to optimizing lipid digestion and absorption, humanized MFG regulates multiple signaling pathways related to neurodevelopment, promoting neuroplasticity and cognitive function. Specifically, humanized MFG modulates neuronal differentiation, enhances the structural integrity of hippocampal neurons, and regulates various neurodevelopment‐related signaling pathways, thereby promoting neurodevelopment in pups. Additionally, it indirectly improves cognitive function by modulating the gut microbiota. This study provides crucial theoretical support for the application of humanized MFG in infant formula and offers innovative solutions for the design and optimization of functional lipids.

## 4 Experimental Section

### Ethical Statement

This study complied with all relevant ethical regulations. All animal experiments were approved by the Animal Experimentation Ethics Committee of Ocean University of China (SPXY2023121101 and SPXY2024041801).

Human milk samples were collected from healthy volunteers with approval from the Human Medical Ethics Committee of Ocean University of China (OUC‐HM‐2024‐24). Informed consent was obtained from all study participants before sample collection.

### MFGM Isolation and Extraction of Its Phospholipids

MFGM separation was carried out following a previously described method.^[^ ^15^, ^52^ ^]^ Raw milk was centrifuged at 5000 × g for 30 min at 4 °C to separate the cream. The collected cream was washed with three volumes of phosphate‐buffered saline (PBS, 0.1 mol L^−1^, pH 6.8) to isolate the MFG. MFG were then disrupted using an ultrasonic homogenizer under the following conditions: 30 cycles of 5 s each, at a frequency of 20 Hz, with 2‐s intervals between cycles. An equal volume of PBS was added to the disrupted MFG, and the mixture was stirred slowly in a 45 °C water bath until a uniform, melted solution was obtained. The mixture was then centrifuged at 1000 × g for 10 min at 25 °C, and the lower‐phase liquid was collected. The isolated milk fat in the upper phase was set aside for later use. The lower cream phase was collected by centrifuging at 1000 × g for 10 min at 25 °C. The pH of the lower‐phase liquid was adjusted to 4.8 using 0.1 mol L^−1^ hydrochloric acid and allowed to stand for 30 min. The mixture was then centrifuged at 3000 × g for 15 min, and the lower liquid phase was collected. The pH of this liquid was adjusted to 6.8 using 0.1 mol L^−1^ sodium hydroxide and freeze‐dried to obtain MFGM.

To extract MFGM phospholipids, 10 volumes of chloroform/methanol (2:1) were added to the freeze‐dried MFGM powder and left overnight. The solution was centrifuged at 7000 × g for 20 min at 4 °C, and the organic phase was dehydrated using a vacuum evaporator at 40 °C to obtain MFGM lipids. Ten volumes of acetone were added to the MFGM lipids and left overnight. The precipitate was recovered by centrifugation at 1260 × g for 5 min and then dissolved in 10 volumes of diethyl ether. After standing overnight, the solution was centrifuged at 1680 × g for 10 min to precipitate the MFGM phospholipids.

### Cell Culture and Treatment

The isolation and culture of NPC were performed following a previously described method, with slight modifications.^[^ ^18^, ^53^ ^]^ Specific pathogen‐free (SPF) female Sprague‐Dawley (SD) rats at gestational days 14‐17 were purchased from Beijing Vital River Laboratory Animal Technology Co., Ltd. (Beijing, China). The rats were provided unrestricted access to food and water for three days before the experiment. Hippocampal NPC cultures were performed using brain tissues from fetal rats at embryonic days 18‐20 (Figure 1a). The fetal rats were euthanized, and the cerebral cortex was dissected. After carefully removing the meninges, the hippocampus was isolated from the cortex. The hippocampal tissue was chopped into small pieces and transferred to a 0.25% trypsin solution to dissociate the cortical tissue into a cell suspension. The resulting suspension was gently triturated using a pipette tip in a pre‐warmed proliferation medium. The cell suspension was filtered through a cell strainer and centrifuged at room temperature. The resulting cell pellet was resuspended in pre‐warmed proliferation medium (DMEM‐F12 supplemented with 10 ng mL^−1^ epidermal growth factor (EGF), 10 ng mL^−1^ fibroblast growth factor (FGF), 2% B‐27, 1% penicillin/streptomycin, and 2 mM L‐glutamine) and seeded in six‐well plates at a density of 1 × 10^5^ cells per well for suspension culture at 37 °C in a humidified atmosphere with 5% CO_2_. Under these proliferation conditions, the cells were allowed to form floating neurospheres for 7 days (Figure 1a). The effects of humanized MFGM‐PL on cell viability were assessed using the CCK8 assay. On day 0 of in vitro culture, MFGM‐PL and humanized MFGM‐PL were added to the medium. Neurospheres were observed under an inverted microscope using bright‐field imaging on days 2, 4, and 7. At least five images were captured for each condition, and neurosphere diameters were quantified using Image J software.

To explore the effects of MFGM‐PL and humanized MFGM‐PL on hippocampal NPC differentiation, cells were seeded in six‐well plates at a density of 5×10^5^ cells per well. After 7 days in the proliferation medium, the culture was transitioned to the differentiation medium (DMEM‐F12 supplemented with 2% B‐27, 1% FBS, 1% penicillin/streptomycin, 2 mM L‐glutamine, and 33 mM D‐glucose). After 2 days of differentiation, the cells were treated with 30 µg mL^−1^ of either MFGM‐PL or humanized MFGM‐PL for 5 days (Figure 1a). After 5 days of exposure to MFGM‐PL and humanized MFGM‐PL, the proportions of βIII‐tubulin and GFAP were analyzed.

### Immunofluorescence Staining

For immunofluorescence analysis of βIII‐tubulin and GFAP in hippocampal neurons, tissue slides were incubated overnight at 4 °C in a humidified dark chamber with either mouse monoclonal anti‐beta III tubulin (ab78078, Abcam; dilution 1:300) or rabbit monoclonal anti‐GFAP antibody (ab33922, Abcam; dilution 1:300). After primary antibody incubation, the slides were incubated at room temperature for 1 h with either Alexa Fluor 594 donkey anti‐mouse (A‐21207, Thermo; dilution 1:200) or Alexa Fluor 488 donkey anti‐rabbit (A‐21202, Thermo; dilution 1:200). Subsequently, the slides were rinsed with PBS and stained with DAPI for 10 min. Fluorescent images were acquired using a CLSM (A1R HD25, Nikon Corporation, Tokyo, Japan). Fluorescence intensity was quantitatively analyzed using ImageJ software (version 1.53e).

### RNA‐seq and Functional Enrichment Analysis

RNA extraction and sequencing were performed using a previously described method^[^ ^54^ ^]^ with slight modifications. Total RNA was extracted using the Trizol method, and RNA concentration was measured using a NanoDrop spectrophotometer. RNA integrity was assessed via 1% agarose gel electrophoresis, and high‐quality RNA samples were selected for library construction.

Library construction and sequencing: mRNA was enriched using Oligo‐dT primers and reverse‐transcribed into cDNA using SuperScript II reverse transcriptase (200 U) in a buffer containing template‐switching oligonucleotides (100 µM), RNase inhibitor (20 U), and dithiothreitol (100 mM). The resulting cDNA was amplified through 15 PCR cycles. After purification with AMPure XP beads, sequencing libraries were constructed and sequenced on the Illumina NovaSeq 6000 platform using a paired‐end strategy.

Data processing and bioinformatics analysis: Raw sequencing reads were quality‐filtered using Fastp (v0.18.0) with a quality score threshold of Q ≥ 20. Gene expression levels were quantified using RSEM (v1.3.3) and expressed as fragments per kilobase million (FPKM). Differentially expressed genes were identified based on FC ≥ 2 and a false discovery rate (FDR)‐adjusted *P* < 0.05.

Pathway and functional enrichment analysis: KEGG pathway enrichment analysis was performed using ClusterProfiler (v4.8.3), and gene set similarity analysis was conducted using EasierEnrichment (v1.10.0). GSEA was performed using hallmark gene sets from MSigDB, and the results were visualized using Enrichplot (v1.20.3).

### Proteomic Analysis

Proteins were extracted from cells using SDS‐DTT‐Tris (SDT) lysis buffer containing 4% sodium dodecyl sulfate. Protein concentration was determined using the BCA assay, and 20 µg of each sample was subjected to SDS‐PAGE for quality control. Protein digestion was performed using the filter‐aided sample preparation method, followed by desalting with a C18 solid‐phase extraction column.^[^ ^55^ ^]^

Liquid chromatography separation: Peptide separation was performed on a NanoElute system using mobile phase A (0.1% formic acid in water) and mobile phase B (0.1% formic acid in acetonitrile). The gradient elution program was as follows: 0–45 min: 5%–25% B; 45–55 min: 25%–40% B; 55–60 min: 40%–95% B. Chromatographic separation was performed on an EASY‐Spray C18 column (75 µm × 25 cm, 1.9 µm) at a flow rate of 300 nL min^−1^.

Mass spectrometry analysis: Mass spectrometry analysis was conducted using a TimsTOF Pro system in positive ion mode with a spray voltage of 1.5 kV. Data were acquired over an m/z range of 100–1700 using the parallel accumulation–serial fragmentation (PASEF) mode, where each MS^1 spectrum triggered 10 MS/MS events. Collision energy was dynamically adjusted based on the m/z value from 20 to 59 eV.

Data processing and bioinformatics analysis: Raw data were analyzed using MaxQuant with the following parameters^[^ ^56^ ^]^: trypsin digestion (allowing up to two missed cleavage sites); precursor mass tolerance of ±6 ppm; variable modification: oxidation (M); fixed modification: carbamidomethylation of cysteine; and FDR threshold of 1%. KEGG pathway enrichment analysis was performed using KOBAS 3.0.

### Reconstruction of Humanized Milk Fat Globules

To prepare the reconstructed humanized MFG, 40 mL of isolated milk fat was added to 3.2 g of MFGM and mixed with 1 L of PBS (0.01 mol L^−1^, pH 7). The mixture was stirred at 200 rpm in a 45 °C water bath using a magnetic stirrer for 3 h, followed by overnight incubation at 4 °C to ensure complete hydration. The hydration mixture was then sonicated for ≈10 min at a frequency of 20 Hz, using 20 cycles of 5 s each with 3 s intervals between cycles. The aqueous and oil phases were emulsified using a shear emulsifier at 13000 rpm for 10 min. The emulsified sample was preheated to 45 °C and homogenized at 200 bars for 10 cycles using a two‐stage homogenizer to obtain reconstructed S‐MFG.

To construct T‐MFG, which integrates both “MFGM lipid composition remodeling” and “tri‐layer membrane structure reconstruction,” 40 mL of isolated milk fat, 3.2 g of MFGM, SM accounting for 17% of the MFGM phospholipid mass, and 1 L of PBS were mixed and processed as described above to produce S‐MFG. An oil/water column was prepared in a 50 mL centrifuge tube, with the following layers arranged from bottom to top as follows: 5 mL of 0.5 M sucrose, an oil phase comprising 6% soybean oil, 8% coconut oil, 20% sunflower oil, 30% milk lipids, and 36% 1,3‐dioleoyl‐2‐palmitoylglycerol (1.5 mL, containing MFGM phospholipids and SM at 17% of the MFGM phospholipid mass). The oil blend was prepared using the ratio reported in the literature, which was similar to the fatty acid composition of breast milk.^[^ ^57^ ^]^ The column was allowed to stabilize for 30 min to form a lipid monolayer at the water/oil interface. Next, 1.5 mL of S‐MFG was carefully added dropwise to the top of the column and allowed to sit for 20 min. The column was then centrifuged at 9000 × g for 30 min at 25 °C to obtain a triple‐layer vesicle in the lower phase. After removing the upper oil layer, the lower aqueous phase was collected and sonicated to yield the reconstructed T‐MFG.

### Characterization

Microstructural observation of the T‐MFG: To observe the microstructure of the T‐MFG, 20 µL of 1 mg mL^−1^ 16:0 Liss Rhod PE fluorescent probe (810158P, Avanti Polar Lipids) was mixed with 1.5 mL of S‐MFG at a 1:40 (v/v) ratio. Similarly, 5 µL of 1 mg mL^−1^ 18:1 PE CF fluorescent probe (810332P, Avanti Polar Lipids) was mixed with the oil phase in the oil/water column at a 1:40 (v/v) ratio. The prepared T‐MFG was then observed using CLSM.

Microstructural observation of the digested MFG: A 200 µL sample aliquot was used for fluorescence labeling. Nile Red (0.1 mg mL^−1^, 20 µL) was mixed with the sample at a 1:10 (v/v) ratio, and the 18:1 PE CF fluorescent probe (1 mg mL^−1^) was mixed at a 1:40 (v/v) ratio. The mixtures were incubated at room temperature in the dark for 30 min, followed by vortex mixing. The samples were then examined using CLSM.

The particle size distribution of the reconstructed fat globules and HM was determined using a laser particle size analyzer (LT2200E, Linkoptik Instruments Co. Ltd., China). The volume‐weighted mean diameter was measured at 10% obscuration. Zeta potential measurements for the reconstructed fat globules and HM were conducted at 25 °C using the Zetasizer Nano ZS90 (Malvern Instruments, UK). At each time point (0, 1, 2, and 4 h), 15 µL of the reconstructed fat globule samples were diluted 400 times in a buffer solution (20 mM imidazole, 50 mM NaCl, 5 mM CaCl_2_) and analyzed at 25 °C. Results were presented as the mean ± standard deviation based on three measurements per sample.

### Animal Models for Simulated Digestion Experiments

Four pregnant SD rats with a precise gestational age of 15 days were purchased from Beijing Vital River Laboratory Animal Technology Co., Ltd. (Beijing, China). The animals were housed under controlled conditions: a 12‐h light/dark cycle, 50‐60% humidity, and a temperature of 22 °C, with free access to food and water. After parturition (approximately gestational day 21), the day of birth was designated as postnatal day 0 (PD0). To standardize nutritional intake, each litter was culled to 10 pups. On PD2, cross‐fostering was conducted to minimize litter‐specific effects: pups born on the same day were pooled, gently mixed, and randomly reassigned to lactating foster dams using a random number table. A total of 48 healthy pups were selected and raised by their respective foster dams until PD14. On PD14, pups were randomly assigned to one of four dietary treatment groups (n = 12 per group) using computer‐generated randomization (Excel RAND function). The groups included human milk (HM), single‐layer milk fat globule (S‐MFG), tri‐layer milk fat globule (T‐MFG), and a non‐reconstructed control (Con) (Figure 2d). The Con group was prepared by mixing 40 mL of isolated milk fat and 3.2 g of MFGM with 1 L of PBS, followed by thorough stirring. Before oral gavage, pups were fasted for 4 h to ensure an empty stomach and deprived of water for 2 h. Each pup received 1.25 mL of the assigned test solution by oral gavage, corresponding to ≈25% of body weight based on an average weight of 30 g. Sampling was performed at 30 min, 1 h, 2 h, and 4 h following oral gavage. At each time point, three pups per group were euthanized, and blood samples were collected via retro‐orbital bleeding. The stomach and small intestine were excised, rinsed, and gently blotted dry. Gastric and intestinal contents were collected and immediately processed to minimize enzymatic degradation. To inhibit post‐sampling enzymatic activity, a protease inhibitor cocktail (0.4 mM Aprotinin, 25 mM Bestatin, 7.5 mM E64, and 10 mM Leupeptin in H_2_O; P1009, Beyotime, Shanghai, China) and a lipase inhibitor (0.2 mL containing 0.4 mM Orlistat; O4139‐25MG, Sigma‐Aldrich, Shanghai, China) were added to each sample. All biological samples were rapidly frozen in liquid nitrogen and stored at ‐80°C until further analysis. For confocal microscopy, the gastric and intestinal contents were mixed with 0.1 mg mL^−1^ Nile Red at a 1:10 (v/v) ratio and 1 mg mL^−1^ PE CF fluorescent probe at a 1:40 (v/v) ratio. The mixture was incubated at room temperature in the dark for 30 min, vortexed, and then observed under a laser confocal microscope to examine the state of the digesta.

### Sample Preparation and Lipidomics Analysis

Sample preparation: After thawing at 4 °C, 20 mg of each sample was weighed and transferred into an eppendorf tube containing steel beads. Then, 200 µL of pre‐cooled butanol/methanol (1:1, containing 10 mmol L^−1^ ammonium formate) was added to the tube. The mixture was vortexed thoroughly and subjected to ultrasonic extraction in an ice bath for 60 min. After centrifugation at 13000 × g for 10 min at 4 °C, the supernatant was collected. Quality control (QC) samples were prepared by pooling equal volumes of the supernatant from each group.

Chromatographic conditions: Ultra‐high‐performance liquid chromatography (UHPLC) was performed using a Nexera LC‐30A system equipped with a Hypersil GOLD C18 column (2.1 × 100 mm, 3 µm). The column temperature was maintained at 40°C, and the flow rate was set at 0.3 mL min^−1^. The mobile phase consisted of solvent A (acetonitrile/water, 4:6 v/v, containing 0.77 g L^−1^ ammonium formate) and solvent B (acetonitrile/isopropanol, 1:9 v/v). The gradient elution program was as follows: 0.1‐4 min: 10%‐40% B; 4‐15 min: 40%‐48% B; 15‐17 min: 48%‐60% B; 17.5‐18.5 min: 60%‐72% B; 18.5‐26 min: 72% B; 26‐26.1 min: 72%‐10% B; 26.1‐30 min: 10% B.

Mass spectrometry conditions: Lipid detection was performed using a Q Exactive Plus mass spectrometer operating in both positive and negative electrospray ionization (ESI) modes. The spray voltages were set to 3.0 kV for the positive mode and 3.5 kV for the negative mode. The capillary temperature was maintained at 350°C. The sheath gas and auxiliary gas flows were set to 45 and 15 arbitrary units (arb), respectively. The scan range was set to m/z 200‐1500.

### Sample Preparation and Metabolomics Analysis

Sample preparation: After thawing, 20 mg of each sample was mixed with 400 µL of pre‐cooled methanol, vortexed, and subjected to ultrasonic extraction in an ice bath for 20 min. The sample was then incubated at ‐20 °C for 1 h, followed by centrifugation at 16000 × g for 20 min at 4 °C. The resulting supernatant was collected and dried under a vacuum. The residue was reconstituted in 100 µL methanol‐water (1:1, v/v), centrifuged, and the supernatant was used for subsequent analysis.

Chromatographic separation: UHPLC was performed using a Nexera LC‐30A system equipped with an ACQUITY UPLC® HSS T3 column (2.1 × 100 mm, 1.8 µm). The column temperature was maintained at 40 °C, and the flow rate was maintained at 0.3 mL min^−1^. The mobile phase consisted of solvent A (0.1% formic acid in water) and solvent B (0.1% formic acid in acetonitrile). The gradient elution program was as follows: 0–2 min: 0% B; 2–6 min: 0%‐48% B; 6–10 min: 48%‐100% B; 10–12 min: 100% B; 12–12.1 min: 100%‐0% B; 12.1–15 min: 0% B.

Mass spectrometry conditions: Metabolite detection was performed using a Q Exactive Plus mass spectrometer operating in both positive and negative ESI modes. The spray voltage was set to 3.8 kV for positive mode and 3.2 kV for negative mode. The capillary temperature was set to 320 °C. The sheath gas and auxiliary gas flow rates were set to 30 and 5 arb, respectively. The scan range was set to m/z 75‐1050. The resolution for MS1 was 70000, with an automatic gain control (AGC) target of 3 × 10⁶. For MS^2^ acquisition, a resolution of 17500 was used, with higher‐energy collisional dissociation (HCD) applied at energies of 20, 30, and 40 eV and an isolation window of 2 m/z.

### Animal Models for Cognitive Experiments

Five SD pregnant rats with an exact gestational age of 15 days were purchased from Beijing Vital River Laboratory Animal Technology Co., Ltd. (Beijing, China). Animals were housed under controlled conditions (12‐h light/12‐h dark cycle, 50‐60% relative humidity, 22 °C) with food and water available ad libitum. All experimental procedures were approved by the Laboratory Animal Ethics Committee of the College of Food Science and Engineering, Ocean University of China (SPXY2023121101). After parturition, designated as postnatal day 0 (PD0), each litter was culled to 10 pups to standardize litter size and ensure consistent nutritional access. On PD2, cross‐fostering was conducted by pooling all pups born on the same day, gently mixing them, and randomly reassigning them to lactating dams using a random number table to minimize litter‐specific effects and equalize maternal care. On PD5, a total of 40 healthy pups were randomly assigned to one of four dietary groups (n = 10 per group) using computer‐generated randomization (Excel RAND function):

1. Breastfed group (BF): Pups were reared by lactating dams and returned to the cages every 4 h for 30 min of nursing.
2. Formula‐fed group (FM): Pups received formula prepared from defatted milk and isolated milk fat, following the method described for S‐MFG preparation in the “Reconstruction of humanized milk fat globules” section.
3. Formula supplemented with single‐layer MFG group (FM‐S‐MFG): Formula was prepared from defatted milk, isolated milk fat, and MFGM, following the method described for S‐MFG preparation in “Reconstruction of humanized milk fat globules” Section.
4. Formula supplemented with tri‐layer MFG group (FM‐T‐MFG): Formula was prepared from defatted milk, isolated milk fat, MFGM, and supplemented with SM at 17% of the MFGM phospholipid mass, following the method described for T‐MFG preparation in “Reconstruction of humanized milk fat globules” section.

The experimental design is illustrated in Figure 3a. All formulae were transferred into 50 mL sterilized centrifuge tubes and stored at −80 °C until use. Before feeding, the formula was thawed and administered via gavage. From PD 5 to PD 21, pups received a formula volume equivalent to 25% of their body weight every 4 h.

### Assessment of Physical Maturity and Reflex Development

Starting on PD 5, daily measurements of body weight, assessments of physical maturity, and evaluations of reflex development were conducted at 10:00 AM. The age of maturity was defined as the day a specific characteristic or reflex was first observed. The following reflex tests were performed:

- Eye‐opening: The time required for the pups to open their eyes was recorded. The maturity day was marked as the first day both eyes of the pups were fully opened.
- Geotaxis test: The pups were placed head‐down on a 20° incline. The maturity day was marked when the pups stood upright (180° from the starting position) within the 90‐s testing period.
- Cliff avoidance: The pups were placed at the edge of a table to assess their ability to avoid falling off within 30 s. The maturity day was defined as the first day the pups retracted their heads and forepaws back from the edge within this time frame.
- Tooth eruption: The timing of tooth eruption was recorded. The maturity day was defined as the first day teeth were visibly erupted.
- Grasp reflex: The pup was held vertically with one or more fingers pressing the thumb against the abdomen and chest to keep the pup still while the fingers supported the back and hind limbs. When the claws extended, a cotton swab was gently applied to the forepaws of the pups, and the grasping behavior was observed.
- Eyelid twitch: A cotton swab was gently used to touch the right eyelid. The maturity day was recorded as the first observation of eyelid twitching.

### Histological Staining

Weaning pups were euthanized by cervical dislocation to obtain brain tissue. The tissue was fixed in 4% paraformaldehyde for 48 h, followed by paraffin infiltration and embedding. Continuous 5 µm sections were cut, deparaffinized, and hydrated before undergoing H&E staining. The pathological features of neurons in the CA1, CA2, and CA3 regions of the hippocampus, along with the dentate gyrus, were examined using an optical microscope. The thickness of the neuronal cell layers was measured using ImageJ 1.53e.

### Immunofluorescent Assays

The brain sections from the pups were deparaffinized using xylene and then dehydrated through a gradient of ethanol (100‐75%). The sections were then immersed in a 0.1 mol L^−1^ PBS solution and washed repeatedly for at least 5 min. After dehydration, the sections were placed in an EDTA antigen retrieval buffer and heated to a simmer for 8 min. The sections were then blocked with 3% bovine serum albumin (BSA) for 30 min. After three washes with 0.1 M PBS (pH 7.4), the sections were incubated overnight at 4 °C with primary antibodies (βIII‐tubulin, 1:300; GFAP, 1:200). The next day, the samples were stained with fluorescence‐labeled secondary antibodies at room temperature for 1 h, and the cell nuclei were stained with DAPI. Finally, the sections were mounted using an anti‐fade mounting medium. Samples were observed using an inverted fluorescence microscope (Ni‐E, Nikon Corporation, Tokyo, Japan), and fluorescence intensity was quantified using Image J 1.53.

### 16S rRNA Sequencing Method

Colon content samples were collected from young rats. Total DNA was extracted using the QIAamp Fast DNA Stool Mini Kit (Qiagen, Hilden, Germany) according to the manufacturer's instructions. DNA concentrations were measured using a Nanodrop‐1000 instrument (Thermo Fisher Scientific), and DNA quality was assessed by agarose (0.8%) gel electrophoresis. The full‐length 16S rRNA gene was amplified using universal PCR primers 27F (5'‐AGAGTTTGATCMTGGCTCAG‐3') and 1492R (5'‐ACCTTGTTACGACTT‐3'). The PCR products were purified for library construction and sequenced on the Illumina MiSeq platform. Raw sequencing reads were demultiplexed and trimmed to remove primer and barcode sequences using Trimmomatic (v.0.39). Clean paired‐end reads were merged into tags using FLASH (v.1.2.11), with a minimum overlap of 10 bp. Chimeric reads were removed using USEARCH (v.7.0.1090), and tags were clustered into operational taxonomic units (OTUs) using VSEARCH (v.2.8.1) with a similarity threshold of 97%. OTUs with fewer than three reads in at least two samples, or those detected in ≤ 0.2% of the samples, were filtered out. Taxonomic classification of OTUs was performed using the Greengenes (v.13.5) database and the RDP classifier (v.2.2). Diversity analyses, including α‐diversity (Shannon's index) and β‐diversity (Bray‐Curtis dissimilarity), were performed using mothur (v.1.43.0) and the vegan package in R.

### Simple Jess Capillary‐Based Electrophoresis Immunoblot Assays

Proteins were extracted using RIPA buffer supplemented with a protease and phosphatase inhibitor cocktail, following the manufacturer's instructions.^[^ ^58^ ^]^ Protein concentration was determined using the Pierce BCA Protein Assay Kit (Thermo Fisher Scientific). Protein separation and detection were conducted using the automated Jess capillary‐based electrophoresis system, according to the manufacturer's protocol (Bio‐techne, Protein Simple, Inc.). Total protein concentration was measured using a protein normalization kit (Bio‐techne, Protein Simple, Inc.) and used as a loading control. Simple Western data were analyzed using Compass for SW software.

### Statistical Analysis

Statistical significance was assessed using a two‐tailed unpaired Student's t‐test for comparisons between two groups and one‐way ANOVA with Sidak's multiple comparison test for multi‐group comparisons. A *P* value of less than 0.05 was considered statistically significant. Data are presented as mean  ± standard error of the mean (SEM). Statistical analyses were performed using GraphPad Prism (version 9.5, GraphPad Software, Inc.).

## Conflict of Interest

The authors declare no conflict of interest.

## Author Contributions

S.L.W. and F.Z.Q. performed examples: S.L.W., F.Z.Q., J.H., and J.X.F. performed conceptualization: S.L.W., F.Z.Q., and J.X.F. performed methodology: S.L.W., F.Z.Q., and J.X.F. performed investigation: S.L.W., F.Z.Q., and J.X.F. performed visualization: H.X.Y., Q.H.S., L.W.Z., and K.L. performed supervision: S.L.W. and K.L. wrote—original draft: S.L.W., FZQ, J.H., J.X.F., T.J.L., H.X.Y., Q.H.S., L.W.Z., and K.L. wrote—reviewed and edited.

## Supporting information



Supporting Information

## Data Availability

The data that support the findings of this study are available in the supplementary material of this article.

## References

[1] T. Gura , Science 2014, 345, 747. 25124424 10.1126/science.345.6198.747

[2] C. G. Victora , B. L. Horta , C. L. de Mola , L. Quevedo , R. T. Pinheiro , D. P. Gigante , H. Gonçalves , F. C. Barros , The Lancet Global Health 2015, 3, 199. 10.1016/S2214-109X(15)70002-1 PMC4365917 25794674

[3] K. Israel‐Ballard , J. Cohen , K. Mansen , M. Parker , C. Engmann , M. Kelley , E. Brooks , E. Chatzixiros , D. Clark , L. Grummer‐Strawn , B. Hartmann , S. Kennedy , G. Kent , M. Mwangome , D. Nyirenda , M. T. Perrin , J.‐C. Picaud , P. Reimers , J. Roest , S. Romero‐Maldonado , J. Smith , P. Subrahmanian , A. Sunder‐Plassmann , G. Weaver , P. A. Zambrano , The Lancet Global Health 2019, 7, 1484. 10.1016/S2214-109X(19)30402-4 PMC7613495 31607455

[4] W. Wei , Q. Jin , X. Wang , Prog. Lipid Res. 2019, 74, 69. 30796946 10.1016/j.plipres.2019.02.001

[5] F. A. Soares , B. Salinas , S. Reis , C. Nunes , Trends Food Sci. Technol. 2023, 141, 104209.

[6] A. Berton , S. Rouvellac , B. Robert , F. Rousseau , C. Lopez , I. Crenon , Food Hydrocoll. 2012, 29, 123.

[7] C. Garcia , C. Antona , B. Robert , C. Lopez , M. Armand , Food Hydrocoll. 2014, 35, 494.

[8] Y. Pan , L. Liu , S. Tian , X. Li , M. Hussain , C. Li , L. Zhang , Q. Zhang , Y. Leng , S. Jiang , S. Liang , Food Hydrocoll. 2022, 124, 107290.

[9] Y. Hou , P. Shen , R. Wang , J. Han , Q. Lin , F. Han , W. Liu , Food Hydrocoll. 2023, 142, 108785.

[10] Q. Ma , X. Zhang , X. Li , L. Liu , S. Liu , D. Hao , A. F. M. Bora , K. J. E.‐P. Kouame , Y. Xu , W. Liu , J. Li , Food Res. Int. 2023, 174, 113574. 37986523 10.1016/j.foodres.2023.113574

[11] Y. Sun , S. Ma , Y. Liu , Z. Jia , X. Li , L. Liu , Q. Ma , K. Jean Eric‐parfait Kouame , C. Li , Y. Leng , S. Jiang , Food Hydrocolloids 2023, 134, 108003.

[12] X. Liu , X. Li , B. Xia , X. Jin , Q. Zou , Z. Zeng , W. Zhao , S. Yan , L. Li , S. Yuan , S. Zhao , X. Dai , F. Yin , E. Cadenas , R. H. Liu , B. Zhao , M. Hou , Z. Liu , X. Liu , Cell Metab. 2021, 33, 923. 33651981 10.1016/j.cmet.2021.02.002

[13] E. A. Mayer , K. Nance , S. Chen , Annu. Rev. Med. 2022, 73, 439. 34669431 10.1146/annurev-med-042320-014032

[14] X. Zou , A. H. Ali , S. M. Abed , Z. Guo , Curr. Opin. Food Sci. 2017, 16, 28.

[15] S. Wang , C. De Souza , M. Ramachandran , Y. Luo , Y. Zhang , H. Yi , Z. Ma , L. Zhang , K. Lin , Food Chem. 2023, 422, 136236. 37130453 10.1016/j.foodchem.2023.136236

[16] J. Fontecha , L. Brink , S. Wu , Y. Pouliot , F. Visioli , R. Jiménez‐Flores , Nutrients 2020, 12, 1607 32486129 10.3390/nu12061607 PMC7352329

[17] Á. Luque‐Uría , M. V. Calvo , F. Visioli , J. Fontecha , Food Funct. 2024, 15, 6783. 38828877 10.1039/d4fo00659c

[18] M. Ramosaj , S. Madsen , V. Maillard , V. Scandella , D. Sudria‐Lopez , N. Yuizumi , L. Telley , M. Knobloch , Nat. Commun. 2021, 12, 7362. 34934077 10.1038/s41467-021-27365-7 PMC8692608

[19] A. M. Bond , G.‐l. Ming , H. Song , Cell Stem Cell 2015, 17, 385. 26431181 10.1016/j.stem.2015.09.003 PMC4683085

[20] C. Thum , N. C. Roy , D. W. Everett , W. C. McNabb , Crit. Rev. Food Sci. Nutr. 2023, 63, 87. 34190660 10.1080/10408398.2021.1944049

[21] M. Martini , F. Salari , I. Altomonte , Crit. Rev. Food Sci. Nutr. 2016, 56, 1209. 24915408 10.1080/10408398.2012.758626

[22] J. H. J. Hageman , M. Danielsen , A. G. Nieuwenhuizen , A. L. Feitsma , T. K. Dalsgaard , Int. Dairy J. 2019, 92, 37.

[23] X. Zhou , H. Hadiatullah , T. Guo , Y. Yao , C. Li , X. Wang , J. Agric. Food Chem. 2021, 69, 10194. 34435766 10.1021/acs.jafc.1c04482

[24] R. I. Wilson , R. A. Nicoll , Science 2002, 296, 678. 11976437 10.1126/science.1063545

[25] I. Katona , T. F. Freund , Nat. Med. 2008, 14, 923. 18776886 10.1038/nm.f.1869

[26] N. Sun , J. Chen , D. Wang , S. Lin , Trends Food Sci. Technol. 2018, 80, 199.

[27] C. J. Heyser , Curr. Protoc. Neurosci. 2003, 25, 8.18. 10.1002/0471142301.ns0818s25 18428605

[28] T. Hainmueller , M. Bartos , Nat. Rev. Neurosci. 2020, 21, 153. 32042144 10.1038/s41583-019-0260-z PMC7115869

[29] M. Schverer , S. M. O'Mahony , K. J. O'Riordan , F. Donoso , B. L. Roy , C. Stanton , T. G. Dinan , H. Schellekens , J. F. Cryan , Neurosci. Biobehav. Rev. 2020, 111, 183. 31945391 10.1016/j.neubiorev.2020.01.012

[30] Y. Zhang , G. Wu , Y. Zhang , X. Wang , Q. Jin , H. Zhang , Comprehens. Rev. Food Sci. Food Safety 2020, 19, 1420. 10.1111/1541-4337.12543 33337094

[31] Y. Yuan , J. Zhao , Q. Liu , Y. Liu , Y. Liu , X. Tian , W. Qiao , Y. Zhao , Y. Liu , L. Chen , Food Chem. 2024, 447, 138991. 38520905 10.1016/j.foodchem.2024.138991

[32] A. H. Merrill , Chem. Rev. 2011, 111, 6387. 21942574 10.1021/cr2002917 PMC3191729

[33] L. S. Kalinichenko , E. Gulbins , J. Kornhuber , C. P. Müller , Prog. Lipid Res. 2022, 86, 101162. 35318099 10.1016/j.plipres.2022.101162

[34] P. Baloni , M. Arnold , L. Buitrago , K. Nho , H. Moreno , K. Huynh , B. Brauner , G. Louie , A. Kueider‐Paisley , K. Suhre , A. J. Saykin , K. Ekroos , P. J. Meikle , L. Hood , N. D. Price , M. Arnold , C. Blach , R. Kaddurah‐Daouk , M. Doraiswamy , S. Mahmoudiandehkordi , K. Welsh‐Bohmer , B. Plassman , J. Krumsiek , R. Batra , A. Saykin , J. Yan , S. L. Risacher , P. Meikle , T. Wang , A. Ikram , et al., Commun. Biol. 2022, 5, 1074. 36209301 10.1038/s42003-022-04011-6 PMC9547905

[35] C. Kranz , C. Jungeblut , J. Denecke , A. Erlekotte , C. Sohlbach , V. Debus , H. G. Kehl , E. Harms , A. Reith , S. Reichel , H. Gröbe , G. Hammersen , U. Schwarzer , T. Marquardt , The Am. J. Human Genet. 2007, 80, 433. 17273964 10.1086/512130 PMC1821118

[36] J. Penney , L.‐H. Tsai , Sci. Signaling 2014, 7, re12. 10.1126/scisignal.aaa0069 25492968

[37] A. Nott , J. Cheng , F. Gao , Y.‐T. Lin , E. Gjoneska , T. Ko , P. Minhas , A. V. Zamudio , J. Meng , F. Zhang , P. Jin , L.‐H. Tsai , Nat. Neurosci. 2016, 19, 1497. 27428650 10.1038/nn.4347 PMC5083138

[38] J. Tornberg , G. P. Sykiotis , K. Keefe , L. Plummer , X. Hoang , J. E. Hall , R. Quinton , S. B. Seminara , V. Hughes , G. Van Vliet , S. Van Uum , W. F. Crowley , H. Habuchi , K. Kimata , N. Pitteloud , H. E. Bülow , Proc. Natl. Acad. Sci. USA 2011, 108, 11524. 21700882 10.1073/pnas.1102284108 PMC3136273

[39] J. F. Cryan , K. J. O'Riordan , C. S. M. Cowan , K. V. Sandhu , T. F. S. Bastiaanssen , M. Boehme , M. G. Codagnone , S. Cussotto , C. Fulling , A. V. Golubeva , K. E. Guzzetta , M. Jaggar , C. M. Long‐Smith , J. M. Lyte , J. A. Martin , A. Molinero‐Perez , G. Moloney , E. Morelli , E. Morillas , R. O'Connor , J. S. Cruz‐Pereira , V. L. Peterson , K. Rea , N. L. Ritz , E. Sherwin , S. Spichak , E. M. Teichman , M. van de Wouw , A. P. Ventura‐Silva , S. E. Wallace‐Fitzsimons , et al., Physiol. Rev. 2019, 99, 1877. 31460832 10.1152/physrev.00018.2018

[40] E. Schneider , K. J. O'Riordan , G. Clarke , J. F. Cryan , Nat. Metabol. 2024, 6, 1454. 10.1038/s42255-024-01108-6 39174768

[41] T. C. Fung , H. E. Vuong , C. D. G. Luna , G. N. Pronovost , A. A. Aleksandrova , N. G. Riley , A. Vavilina , J. McGinn , T. Rendon , L. R. Forrest , E. Y. Hsiao , Nat. Microbiol. 2019, 4, 2064. 31477894 10.1038/s41564-019-0540-4 PMC6879823

[42] E. Aaldijk , Y. Vermeiren , Ageing Res. Rev. 2022, 75, 101556. 34990844 10.1016/j.arr.2021.101556

[43] S. J. B. Dunham , J. Avelar‐Barragan , J. A. Rothman , E. D. Adams , G. Faraci , S. Forner , S. Kawauchi , A. J. Tenner , K. N. Green , F. M. LaFerla , G. R. MacGregor , M. Mapstone , K. L. Whiteson , Alzheimer's & Dementia 2024, 20, 4935. 10.1002/alz.13794 PMC11247698 38572865

[44] R. T. Liu , A. D. Rowan‐Nash , A. E. Sheehan , R. F. L. Walsh , C. M. Sanzari , B. J. Korry , P. Belenky , Brain, Behav., Immun. 2020, 88, 308. 32229219 10.1016/j.bbi.2020.03.026 PMC7415740

[45] D. Radjabzadeh , J. A. Bosch , A. G. Uitterlinden , A. H. Zwinderman , M. A. Ikram , J. B. J. van Meurs , A. I. Luik , M. Nieuwdorp , A. Lok , C. M. van Duijn , R. Kraaij , N. Amin , Nat. Commun. 2022, 13, 7128. 36473852 10.1038/s41467-022-34502-3 PMC9726982

[46] W. Zhang , Y. Guo , Y. Cheng , W. Yao , H. Qian , Int. J. Biol. Macromol. 2023, 225, 974. 36402384 10.1016/j.ijbiomac.2022.11.160

[47] E. Biagi , L. Nylund , M. Candela , R. Ostan , L. Bucci , E. Pini , J. Nikkïla , D. Monti , R. Satokari , C. Franceschi , P. Brigidi , W. De Vos , PLoS One 2010, 5, 10667. 10.1371/journal.pone.0010667 PMC2871786 20498852

[48] M. Chini , I. L. Hanganu‐Opatz , Trends Neurosci. 2021, 44, 227. 33246578 10.1016/j.tins.2020.10.017

[49] M. S. Blumberg , K. E. Adolph , Trends Cogn. Sci. 2023, 27, 696. 37321923 10.1016/j.tics.2023.05.008 PMC10528200

[50] A. Loughman , A.‐L. Ponsonby , M. O'Hely , C. Symeonides , F. Collier , M. L. K. Tang , J. Carlin , S. Ranganathan , K. Allen , A. Pezic , R. Saffery , F. Jacka , L. C. Harrison , P. D. Sly , P. Vuillermin , eBioMedicine 2020, 52, 102640. 32062351 10.1016/j.ebiom.2020.102640 PMC7016366

[51] Y. E. Borre , G. W. O'Keeffe , G. Clarke , C. Stanton , T. G. Dinan , J. F. Cryan , Trends Mol. Med. 2014, 20, 509. 24956966 10.1016/j.molmed.2014.05.002

[52] S. Wang , P. Wang , X. Ma , F. Qiao , Z. Zhang , J. Li , H. Yi , C. De Souza , L. Zhang , K. Lin , Food Chem. 2025, 468, 142478. 39700807 10.1016/j.foodchem.2024.142478

[53] C.‐L. Wang , R. Ohkubo , W.‐C. Mu , W. Chen , J. L. Fan , Z. Song , A. Maruichi , P. H. Sudmant , A. O. Pisco , D. B. Dubal , N. Ji , D. Chen , Cell Metab. 2023, 35, 996. 37146607 10.1016/j.cmet.2023.04.012 PMC10330239

[54] Z. Fu , Z. Liu , J. Wang , L. Deng , H. Wang , W. Tang , D. Ni , Biomaterials 2023, 295, 122035. 36764193 10.1016/j.biomaterials.2023.122035

[55] J. R. Wiśniewski , M. Mann , Anal. Chem. 2012, 84, 2631. 22324799 10.1021/ac300006b

[56] P. Kumar , A. M. Goettemoeller , C. Espinosa‐Garcia , B. R. Tobin , A. Tfaily , R. S. Nelson , A. Natu , E. B. Dammer , J. V. Santiago , S. Malepati , L. Cheng , H. Xiao , D. D. Duong , N. T. Seyfried , L. B. Wood , M. J. M. Rowan , S. Rangaraju , Nat. Commun. 2024, 15, 2823. 38561349 10.1038/s41467-024-47028-7 PMC10985119

[57] Q. Ma , X. Zhang , Y. Zhao , X. Li , L. Liu , X. Zhang , Food Hydrocolloids 2024, 157, 110433.

[58] S. Wang , X. Wang , Y. Shan , Z. Tan , Y. Su , Y. Cao , S. Wang , J. Dong , J. Gu , Y. Wang , Cell Stem Cell 2024, 31, 341. 38402618 10.1016/j.stem.2024.01.013

