# Supplementary material for: Phospholipid Remodeling and Tri‐Layer Membrane Reconstruction Mediate Cognitive Effects of Humanized Milk Fat Globules in Neonatal Rats
Source: Adv Sci (Weinh). 2025 Sep 14;12(45):e07926. doi: 10.1002/advs.202507926 (PMC12677656; doi:10.1002/advs.202507926)
Supplement: Supplementary file 1 — Supporting Information [file ADVS-12-e07926-s001.docx]

**Phospholipid Remodeling and Tri-layer Membrane Reconstruction Mediate Cognitive Effects of Humanized Milk Fat Globules in Neonatal Rats**

*Shaolei Wang ^1^, Fengzhi Qiao ^1^, Jian He ^2^, Jianxin Fu ^1^, Tongjie Liu ^1^, Huaxi Yi ^1^, Qinghai Sheng ^3^, Lanwei Zhang ^1^*, Kai Lin ^1^**

1 State Key Laboratory of Marine Food Processing & Safety Control, College of Food Science and Engineering, Ocean University of China, Qingdao 266003, China.

2 National Center of Technology Innovation for Dairy, Hohhot 010000, China.

3 College of Food Science and Technology, Hebei Agricultural University, Baoding 071001, China.

*Corresponding author. Kai Lin, [linkai@ouc.edu.cn](mailto:linkai@ouc.edu.cn); Lanwei Zhang, [zhanglanwei@ouc.edu.cn](mailto:zhanglanwei@ouc.edu.cn)

Supplementary Text


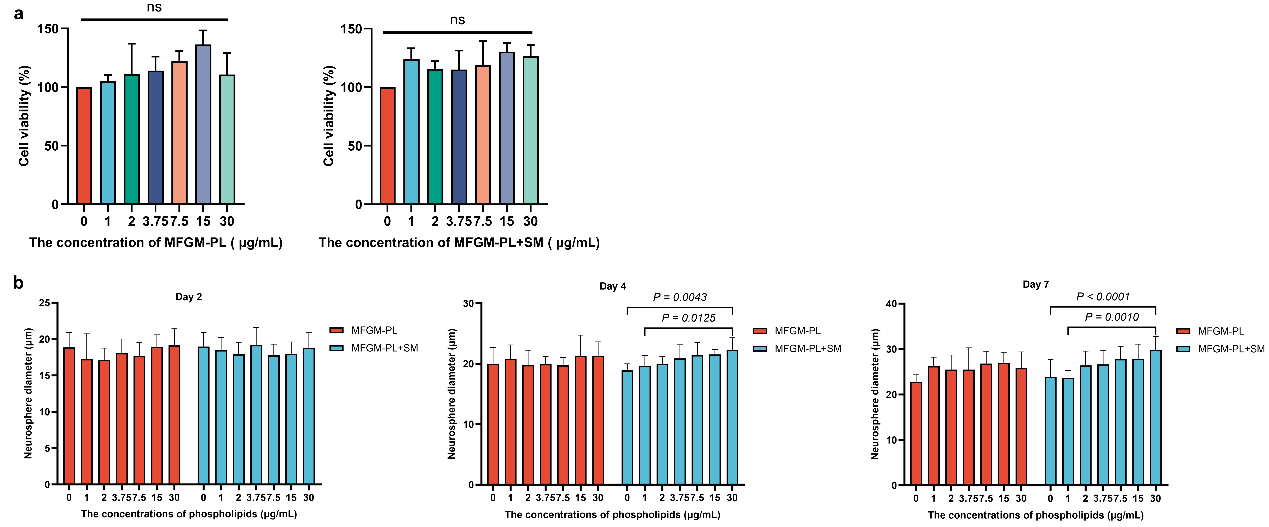


Fig. S1. Effects of humanized milk fat globule membrane phospholipid (MFGM-PL+SM, enriched with sphingomyelin) composition on viability and proliferation of hippocampal neural progenitor cells (NPC). (a) Cytotoxicity assay of hippocampal NPC treated with varying concentrations (0-30 μg/mL) of MFGM-PL or MFGM-PL+SM. NPC were isolated from embryonic day 18-20 rat hippocampi and cultured in proliferation medium for 24 hours prior to treatment. Cell viability was assessed using the Cell Counting Kit-8 (CCK-8) assay. No significant cytotoxicity was observed at any tested concentration. (b) Proliferation assay based on neurosphere diameter. NPC were cultured in suspension and treated with MFGM-PL or MFGM-PL+SM with varying concentrations (0-30 μg/mL). Neurosphere images were captured on days 2, 4, and 7 using bright-field microscopy. Quantification of neurosphere diameter showed significantly enhanced proliferation in the 15 and 30 μg/mL MFGM-PL+SM groups at days 4 and 7. (n=6).


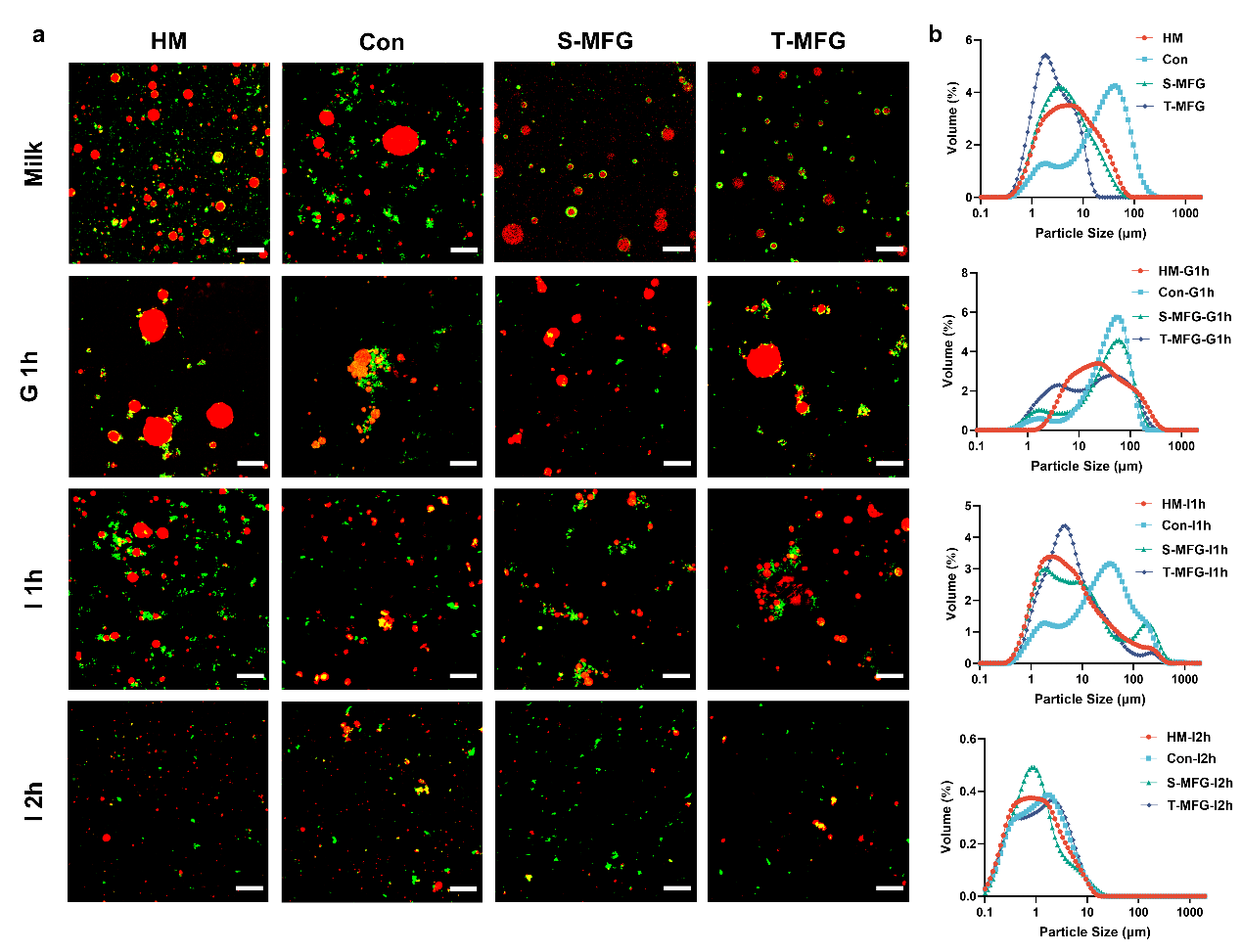


Fig. S2. *In vitro* simulation of infant gastrointestinal digestion of reconstructed humanized milk fat globules (MFG). (a) Microstructure of fat globules before and during digestion. Representative confocal laser scanning microscopy (CLSM) images illustrate the structural changes in fat globules from human milk (HM), control (Con), single-layer milk fat globules (S-MFG), and tri-layer milk fat globules (T-MFG) throughout simulated infant gastrointestinal digestion. Fluorescent probes were used to label triacylglycerols (Nile Red) and phospholipids (18:1 PE CF). T-MFG and HM maintained more intact phospholipid-coated structures during gastric digestion, whereas the Con group exhibited larger aggregates. Scale bar = 20 μm. (b) Particle size distribution of fat globules during digestion. Particle sizes of HM, Con, S-MFG, and T-MFG were measured at different digestion time points (0, 1, 2, and 4 h) using a laser particle size analyzer. T-MFG showed size distribution patterns more closely resembling HM, with minimized flocculation and aggregation during gastric and intestinal phases. (n=3).


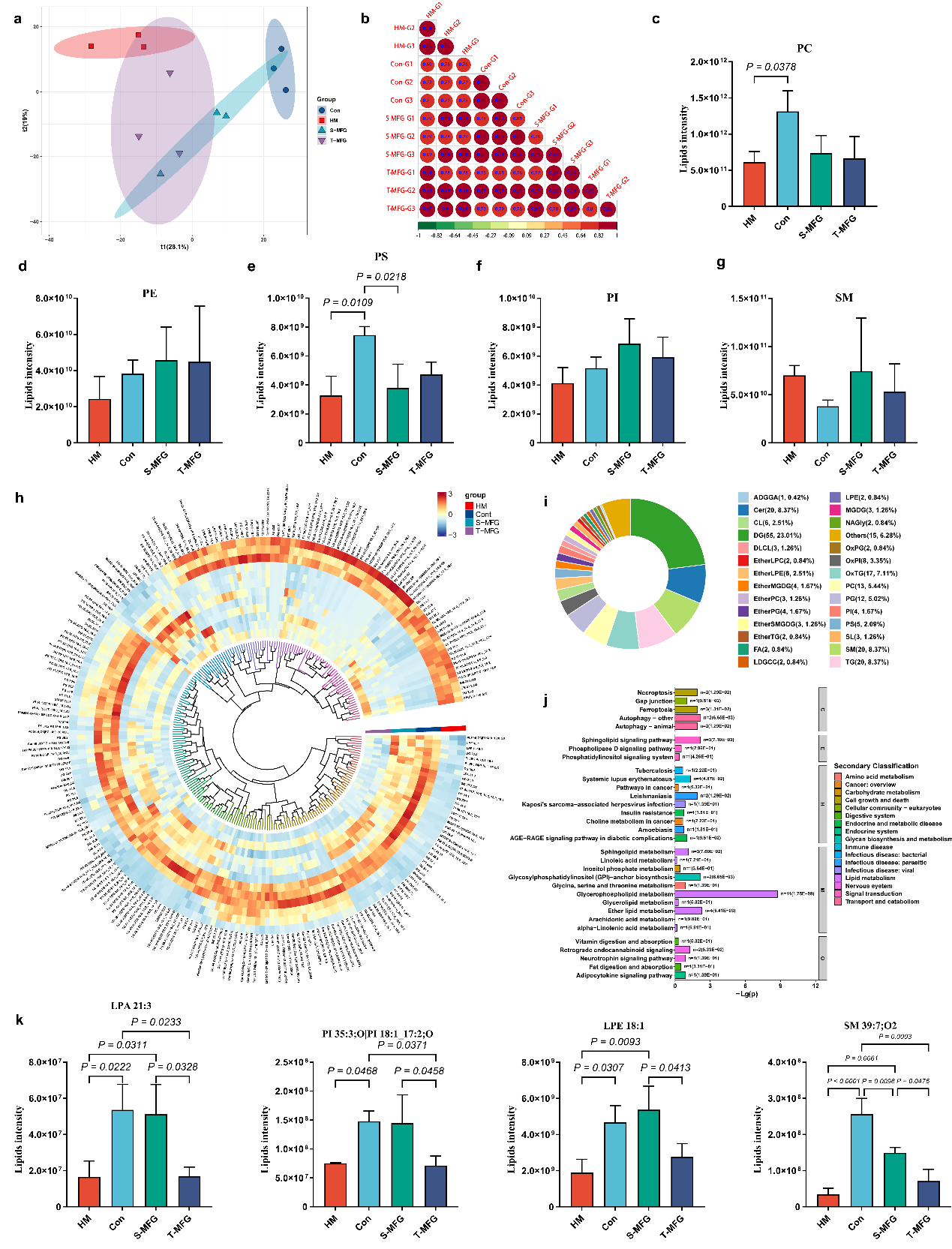


Fig. S3. The effects of humanized milk fat globules (MFG) on lipid composition during gastric digestion in neonatal rats. (a) PLS-DA (Partial Least Squares Discriminant Analysis) illustrating group separations based on lipidomic profiles of gastric contents from four groups: human milk (HM), control (Con), single-layer milk fat globules (S-MFG), and tri-layer milk fat globules (T-MFG). T-MFG clustered closer to HM, suggesting compositional similarity. (b) Correlation matrix analysis of lipid species among groups, showing stronger lipid profile correlations between HM and T-MFG (mean r = 0.87) than between HM and S-MFG (mean r = 0.81). (c-g) Intergroup comparison of major phospholipid classes, including phosphatidylcholine (PC), phosphatidylethanolamine (PE), phosphatidylinositol (PI), phosphatidylserine (PS), and sphingomyelin (SM). Phospholipid profiles in S-MFG and T-MFG were more similar to HM than those in the Con group. (h) Hierarchical clustering heatmap of significantly different lipid species during gastric digestion. (i) Classification of differentially expressed lipids, with diglycerides (DG), ceramides (Cer), sphingomyelin (SM), and triglycerides (TG) being most significantly affected by MFG structure. (j) KEGG pathway enrichment analysis identified key metabolic pathways associated with significantly different lipids, including glycerophospholipid metabolism, and fat/vitamin digestion and absorption. (k) Comparison of significantly different lipid species among groups during gastric digestion stage. (n=3).


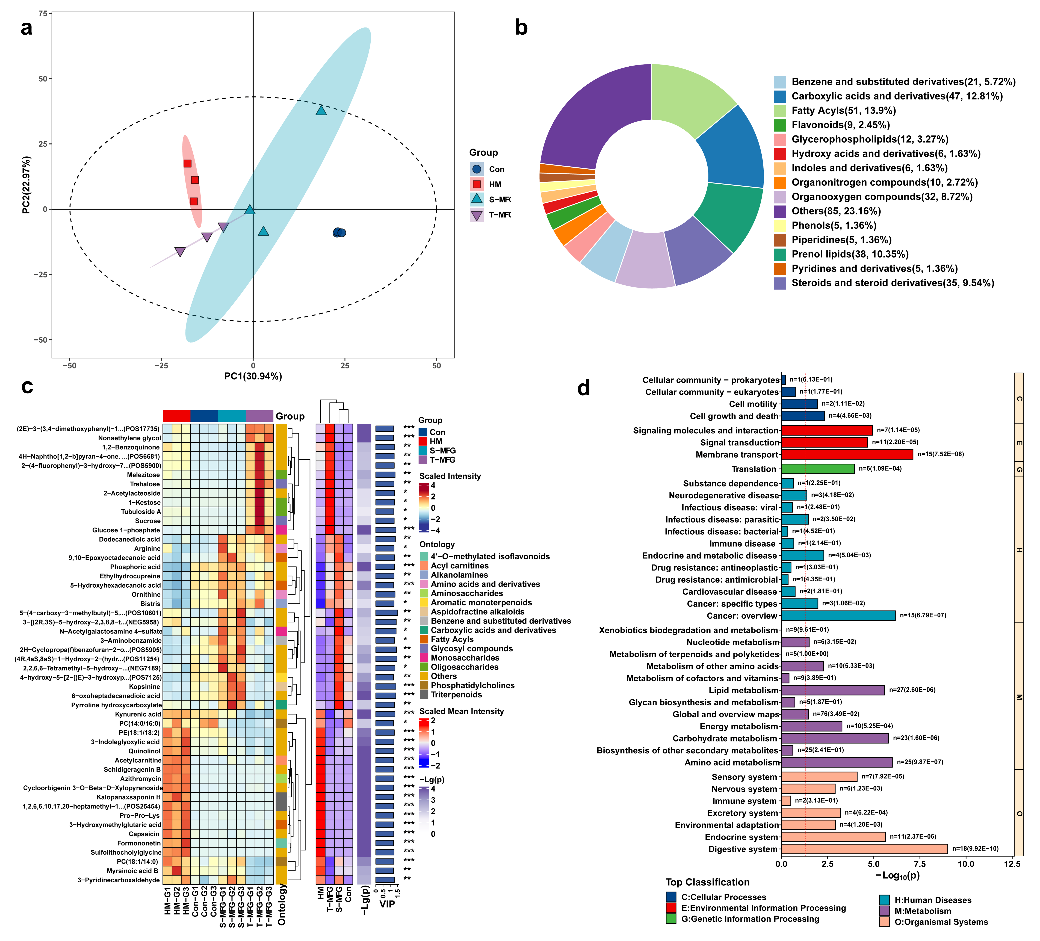


Fig. S4. (a) Principal component analysis (PCA) of metabolomic data from four groups: human milk (HM), control (Con), single-layer milk fat globules (S-MFG), and tri-layer milk fat globules (T-MFG). The PCA plot demonstrates that the metabolite profiles of HM and T-MFG clustered together, indicating high compositional similarity during gastric digestion. (b) Classification of 367 significantly different metabolites identified among the groups based on metabolite class. The most abundant classes included fatty acyls (13.9%), carboxylic acids and derivatives (12.81%), prenol lipids (10.35%), and steroids/steroid derivatives (9.54%). (c) Heatmap cluster analysis of the top 50 significantly different metabolites (based on VIP scores). (d) Pathway enrichment analysis of the significantly altered metabolites, revealing major involvement in lipid metabolism and digestion-associated pathways. (n=3).


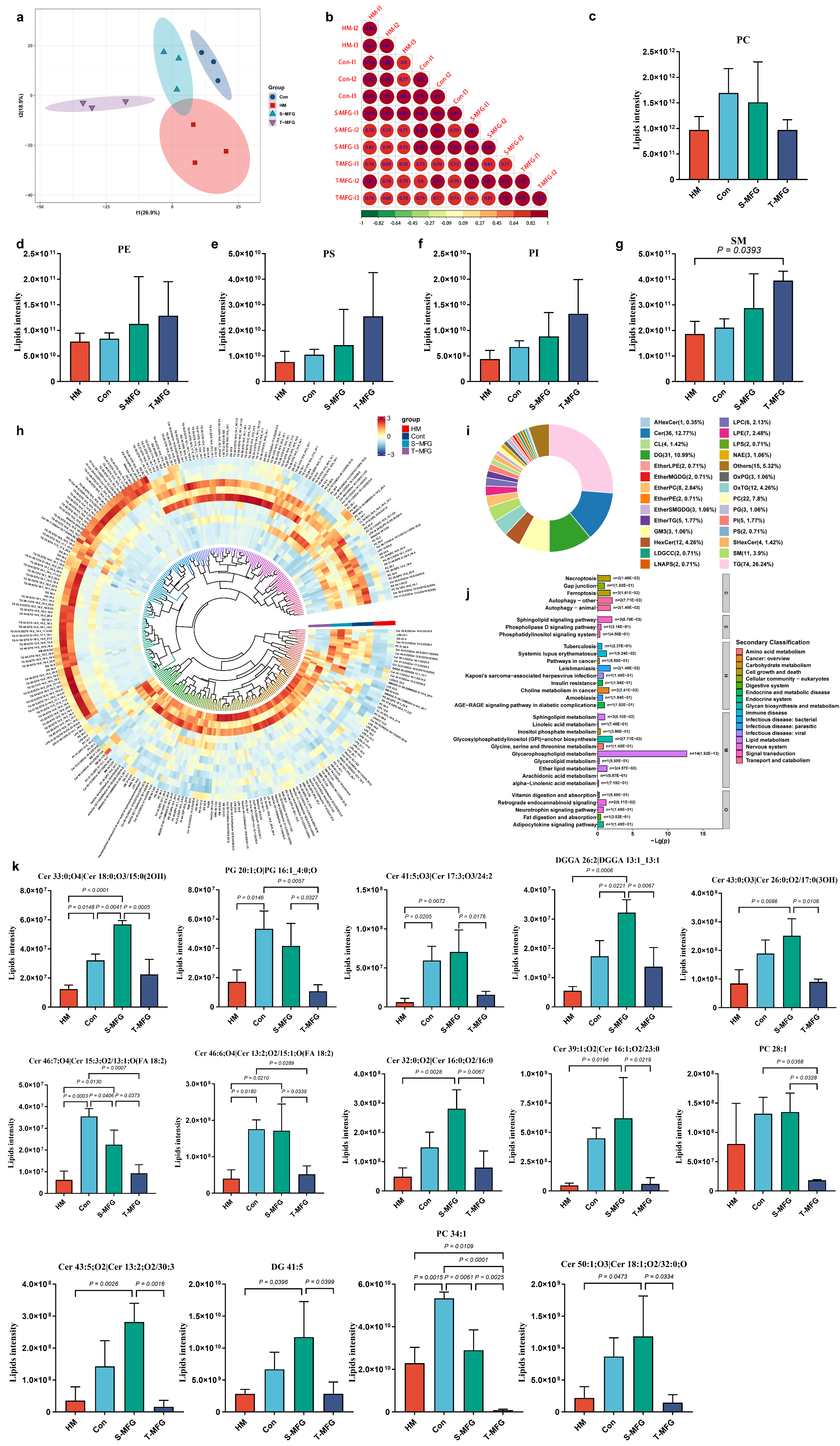


Fig. S5. The effects of humanized milk fat globules (MFG) on lipid composition during intestinal digestion in neonatal rats. (a) Partial least squares discriminant analysis (PLS-DA) of intestinal lipidomic profiles among four dietary groups: human milk (HM), control (Con), single-layer milk fat globules (S-MFG), and tri-layer milk fat globules (T-MFG). HM and T-MFG samples clustered together on the lower side of principal component 2, indicating similar lipid profiles, whereas S-MFG and Con formed a separate cluster. (b) Pairwise correlation analysis of lipid profiles among groups, showing a moderate positive correlation. The average correlation coefficient between T-MFG and HM was 0.76, slightly lower than that between S-MFG and HM (0.79), with all values < 0.8. (c-g) Intergroup comparison of major phospholipid classes, including phosphatidylcholine (PC), phosphatidylethanolamine (PE), phosphatidylinositol (PI), phosphatidylserine (PS), and sphingomyelin (SM). Notably, sphingomyelin (SM) levels in the T-MFG group were significantly higher than those in the HM group during intestinal digestion. (h) Hierarchical clustering heatmap of significantly different lipid species. (i) Classification of major lipid categories among the significantly different lipids. The most abundant classes were triglycerides (TG, 26.24%), ceramides (Cer, 12.77%), diglycerides (DG, 10.99%), and phosphatidylcholines (PC, 7.8%). (j) Metabolic pathway enrichment analysis using KEGG, highlighting the involvement of the differentially abundant lipid species in pathways related to glycerophospholipid metabolism, lipid digestion, and nutrient absorption. (k) Comparison of significantly different lipid species among groups during intestinal digestion stage. (n=3).


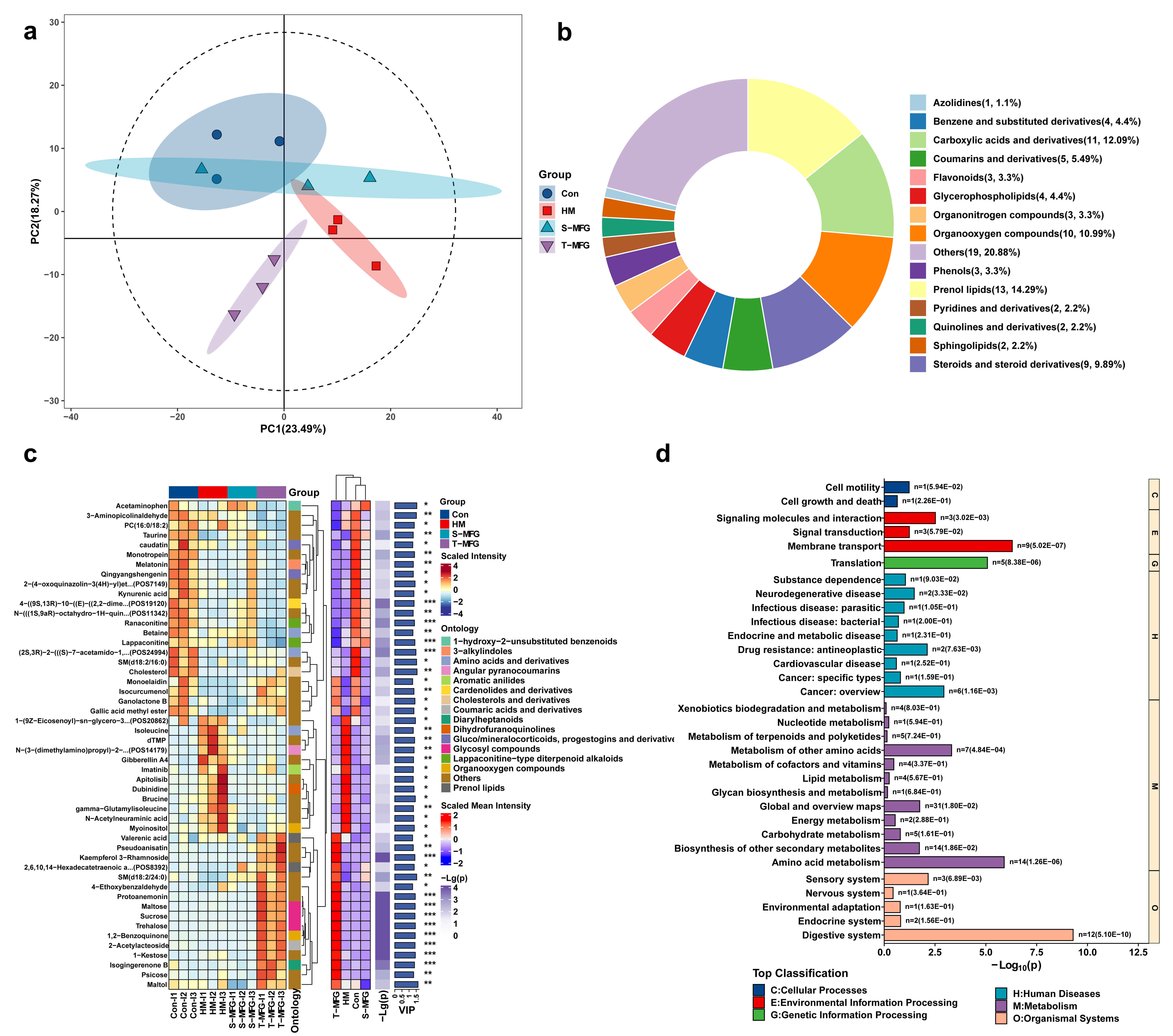


Fig. S6. The effect of humanized milk fat globules (MFG) on the metabolomics during the intestinal digestion phase in neonatal rats. (a) Principal component analysis (PCA) of intestinal metabolite profiles in four feeding groups: human milk (HM), control (Con), single-layer milk fat globules (S-MFG), and tri-layer milk fat globules (T-MFG). The PCA plot shows that HM and T-MFG clustered together along principal component 2, whereas S-MFG and Con formed a separate cluster, suggesting that T-MFG more closely resembles the metabolic profile of HM during intestinal digestion. (b) Classification of 93 significantly different metabolites among groups based on metabolite class. The major metabolite classes were prenol lipids (14.29%) and carboxylic acids and derivatives (12.09%), which are associated with digestion and absorption pathways. (c) Heatmap cluster analysis of the top 50 significantly different metabolites (ranked by VIP scores). (d) Pathway enrichment analysis of the significantly altered metabolites, indicating that the differences were primarily involved in digestion-associated metabolic pathways. (n=3).





Fig. S7. The effects of humanized milk fat globules (MFG) on lipid composition of serum in neonatal rats. (a) Partial least squares discriminant analysis (PLS-DA) of serum lipidomic profiles among four dietary groups: human milk (HM), control (Con), single-layer milk fat globules (S-MFG), and tri-layer milk fat globules (T-MFG). HM and T-MFG samples clustered on the left side of principal component 1, indicating comparable serum lipid compositions. S-MFG samples were near the origin, and Con samples clustered on the right, suggesting a gradation of similarity to HM with respect to MFG structure. (b) Correlation analysis of serum lipid composition among groups. All treatment groups exhibited strong positive correlations with HM (correlation coefficients > 0.8), suggesting general similarity in overall serum lipid profiles. (c-g) Intergroup comparison of major phospholipid classes, including phosphatidylcholine (PC), phosphatidylethanolamine (PE), phosphatidylinositol (PI), phosphatidylserine (PS), and sphingomyelin (SM). No statistically significant differences were observed among the four groups, indicating stable phospholipid distribution in serum. (h) Hierarchical clustering heatmap based on significantly different lipid species. (i) Classification of 226 significantly different lipid species into major lipid categories. The most abundant classes were triglycerides (TG, 39.07%), phosphatidylcholine (PC, 11.16%), oxidized triacylglycerols (OxTG, 6.98%), and phosphatidylinositol (PI, 5.58%). (j) KEGG pathway enrichment analysis of the differential lipid species, revealing involvement in lipid metabolism pathwaysand neurobiological processes. (k) Comparison of significantly different lipid species among groups in rat pup serum. (n=3).


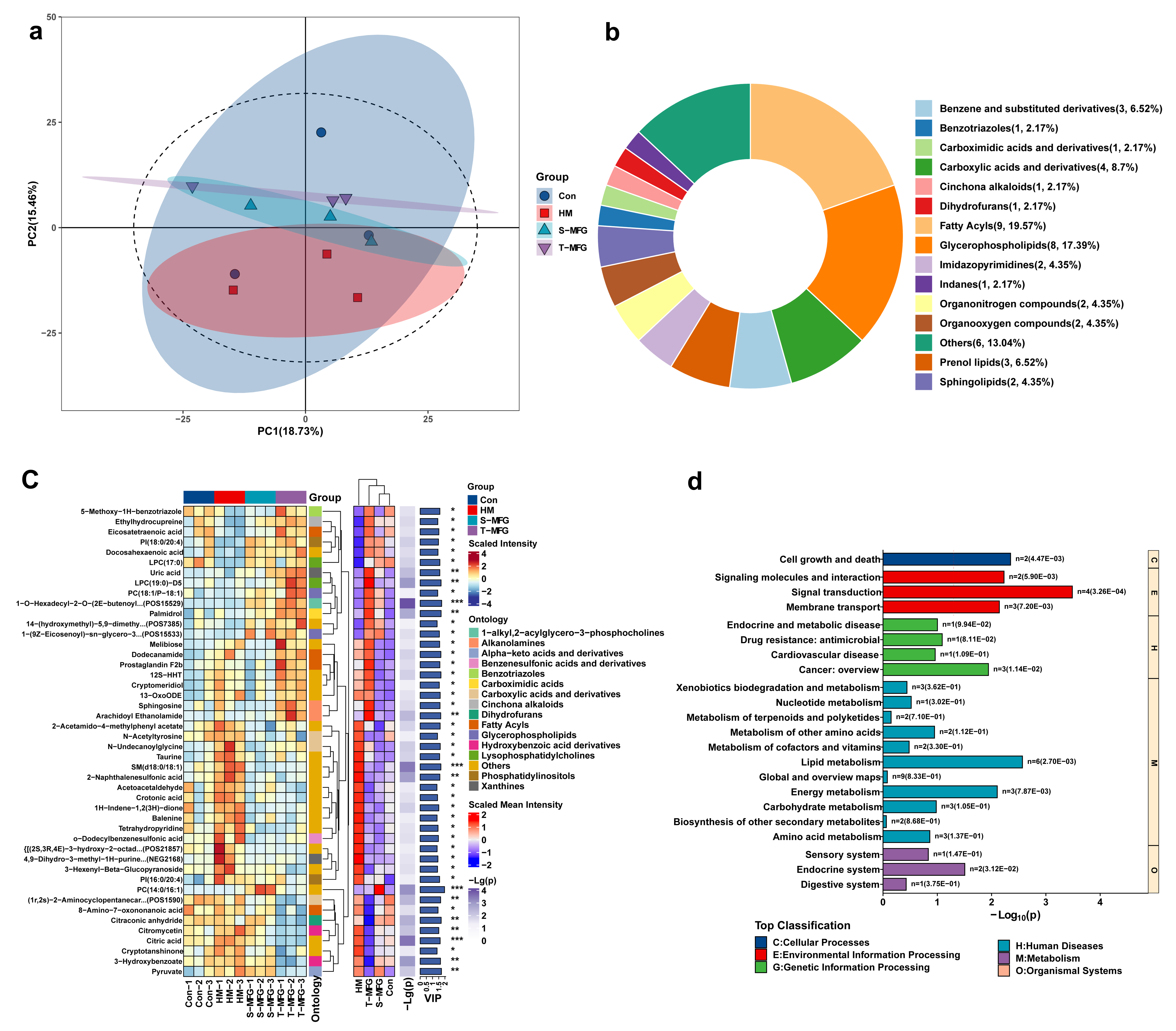


Fig. S8. The effect of humanized milk fat globules (MFG) on the metabolomics of serum in neonatal rats. (a) Principal component analysis (PCA) of serum metabolite profiles across four groups: human milk (HM), control (Con), single-layer milk fat globules (S-MFG), and tri-layer milk fat globules (T-MFG). All samples clustered near the origin of the PCA plot, indicating minimal variation in the overall serum metabolome between groups. (b) Classification of 46 significantly different metabolites detected in serum. Fatty acyls (19.57%) were the most abundant class, followed by glycerophospholipids (17.39%) and carboxylic acid derivatives (8.7%), reflecting subtle differences in systemic lipid metabolism across diets. (c) Heatmap cluster analysis of the top 50 significant metabolites (ranked by VIP scores). (d) Pathway enrichment analysis of differential serum metabolites using the KEGG database, indicating involvement in lipid metabolic processes and signal transduction pathways. (n=3).


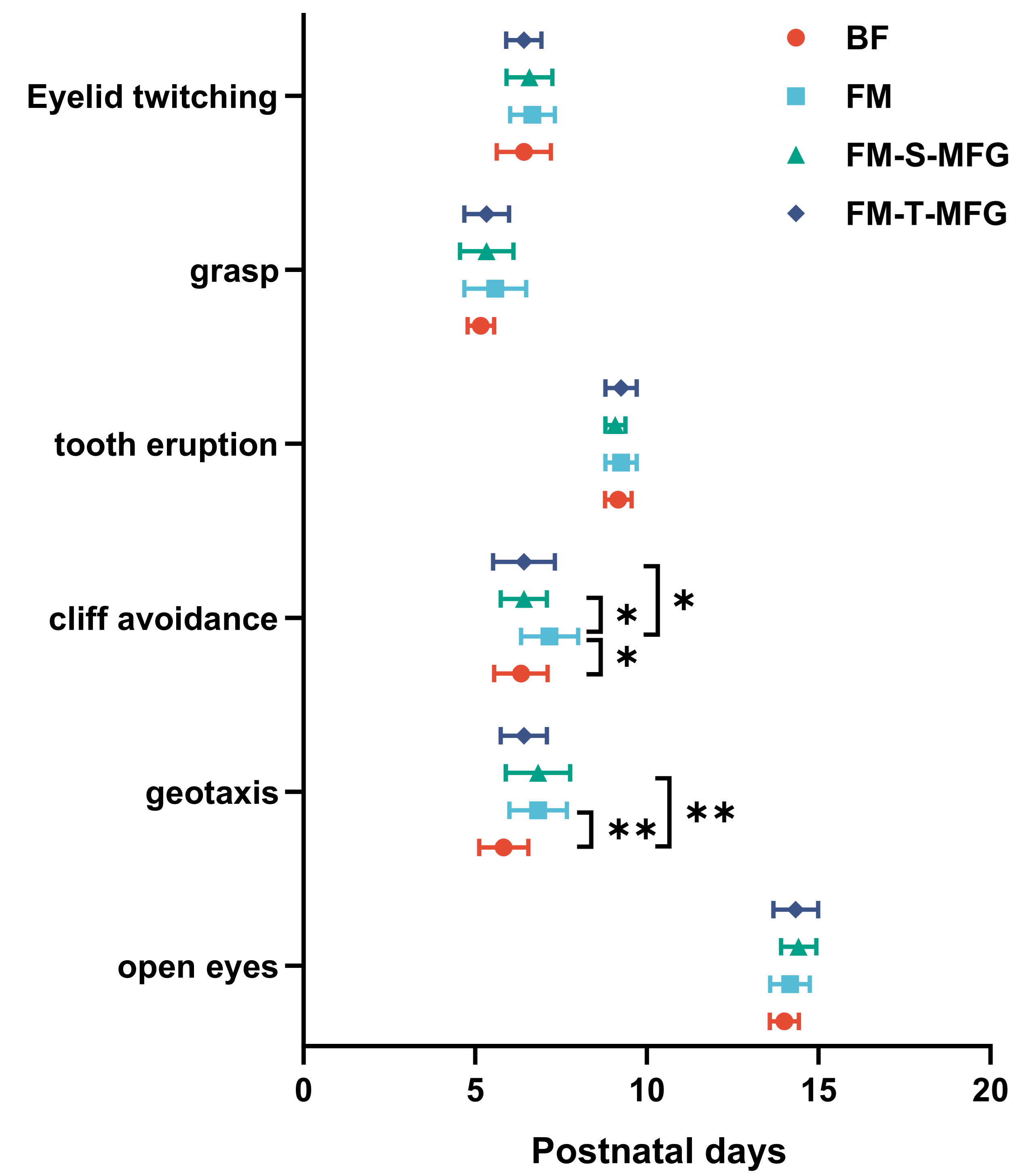


Fig. S9. Evaluation of humanized milk fat globules (MFG) on cognitive and neurodevelopmental reflexes in neonatal rats. To investigate the influence of dietary MFG structure on early neurodevelopment, rat pups were divided into four groups based on feeding regimen: breast-fed group (BF), formula milk group (FM), formula-fed containing S-MFG group (FM-S-MFG), and formula-fed containing T-MFG group (FM-T-MFG). Neurodevelopmental and behavioral maturation was assessed by measuring key reflexes and developmental milestones, including: eyelid twitching, grasp, tooth eruption, cliff avoidance, geotaxis, and open eye (n=10).
